# Exploring biopsychosocial correlates of pregnancy risk and pregnancy intention in women with chronic kidney disease

**DOI:** 10.1007/s40620-023-01610-2

**Published:** 2023-03-27

**Authors:** Elizabeth R. Ralston, Priscilla Smith, Katherine Clark, Kate Wiles, RaDaR CKD Pregnancy Group, Joseph Chilcot, Kate Bramham

**Affiliations:** 1 Department of Women and Children’s Health, School of Life Course Sciences, King’s College London, 5th Floor Addison House, Guy’s Campus, London, SE1 1UL UK; 2 Department of Obstetric Medicine, Bart’s and the London NHS Foundation Trust, London, UK; 3 Department of Psychology, Institute of Psychiatry, Psychology and Neuroscience, King’s College London, London, UK; 4 Department of Renal Medicine, School of Inflammation, Immunology and Mucosal Biology, King’s College London, London, UK

**Keywords:** Pregnancy, CKD, Risk perception, Pregnancy intention

## Abstract

**Introduction:**

Women with Chronic Kidney Disease (CKD) are at increased risk of adverse pregnancy and renal outcomes. It is unknown how women with CKD understand their pregnancy risk. This nine-centre, cross-sectional study aimed to explore how women with CKD perceive their pregnancy risk and its impact on pregnancy intention, and identify associations between biopsychosocial factors and perception of pregnancy risk and intention.

**Methods:**

Women with CKD in the UK completed an online survey measuring their pregnancy preferences; perceived CKD severity; perception of pregnancy risk; pregnancy intention; distress; social support; illness perceptions and quality of life. Clinical data were extracted from local databases. Multivariable regression analyses were performed. Trial registration: NCT04370769.

**Results:**

Three hundred fifteen women participated, with a median estimated glomerular filtration rate (eGFR) of 64 ml/min/1.73m^2 ^(IQR 56). Pregnancy was important or very important in 234 (74%) women. Only 108 (34%) had attended pre-pregnancy counselling. After adjustment, there was no association between clinical characteristics and women’s perceived pregnancy risk nor pregnancy intention. Women’s perceived severity of their CKD and attending pre-pregnancy counselling were independent predictors of perceived pregnancy risk. Importance of pregnancy was an independent predictor of pregnancy intention but there was no correlation between perceived pregnancy risk and pregnancy intention (*r* = − 0.002, 95% CI − 0.12 to 0.11).

**Discussion:**

Known clinical predictors of pregnancy risk for women with CKD were not associated with women’s perceived pregnancy risk nor pregnancy intention. Importance of pregnancy in women with CKD is high, and influences pregnancy intention, whereas perception of pregnancy risk does not.

**Graphical abstract:**

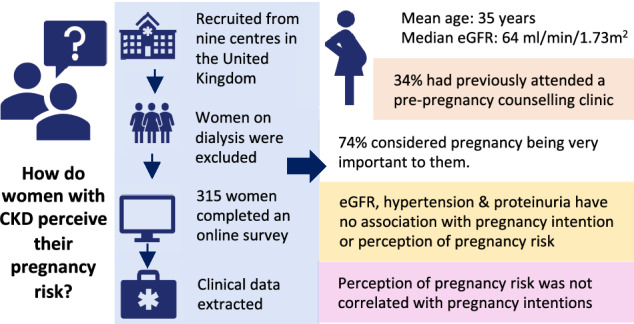

**Supplementary Information:**

The online version contains supplementary material available at 10.1007/s40620-023-01610-2.

## Introduction

Three percent of women of reproductive age are estimated to be affected by Chronic Kidney Disease (CKD) [1], with prevalence anticipated to rise with increasing rates of obesity [2], diabetes [3] and advancing maternal age [4]. Pregnancies with CKD are complicated by increased risk to both mother and baby including superimposed pre-eclampsia [5, 6], preterm birth [6, 7], small for gestational age infants [6, 8], admission to neonatal care [6], and acceleration of CKD progression [5, 6].

Pre-pregnancy counselling clinics are an opportunity to discuss potential pregnancy risk with provision of individualised care and psychological preparation for a complex pregnancy [9, 10]. Pre-pregnancy counselling aims to help set realistic expectations, discuss potential maternal and fetal risk, adjust medication, and optimise timings [9]. It is recommended by the UK Kidney Association and within the Confidential Enquiries into Maternal Deaths in the United Kingdom that women with pre-existing medical conditions are referred for pre-pregnancy counselling by a multidisciplinary team [4, 11]. However, provision of information related to objective risk should not assume that women will perceive the same risk [12–14]. An individual’s perceived risk and perceptions pertaining to their health condition can be used to understand and predict health-related behaviour and decisions [15, 16]. In women without CKD, perceived pregnancy risk has been reported to influence women’s pregnancy-related behaviour and decision making [17, 18] but knowledge of risk perception of pregnancy and how it may influence pregnancy intentions in women with CKD is limited. Improved understanding of how women with CKD perceive pregnancy risk and factors influencing pregnancy intention is likely to enhance risk communication and provision of individualised care.

The aims of this study were to understand how women with CKD perceive pregnancy risk, to examine the relationship between biopsychosocial factors and perception of pregnancy risk and pregnancy intentions, and to understand the relationship between perceived pregnancy risk and pregnancy intention.

## Materials and methods

### Design

This was an online cross-sectional study of women of reproductive age with CKD in the United Kingdom, registered online at ClinicalTrials.Gov (Study ID: NCT04370769, 30.04.2020). Ethical approval was given by the Research Ethics Committee and Health Research Authority (London Bloomsbury Research Ethics Committee, Ref: 20/LO/0257).

### Participants and procedure

Women who had attended routine renal clinics and/or renal pre-pregnancy counselling at nine sites in the United Kingdom between October 2020 to December 2021 were recruited. Women were eligible if they were between 18 to 50 years old, diagnosed with CKD Stages 1–5 according to the Kidney Disease Improving Global Outcomes guidelines [19], had attended a pre-pregnancy or renal clinic within the last 2 years (2018–2021) and English speaking due to lack of validation of several standardised measures in non-English languages. Women were excluded if they were established on haemodialysis or peritoneal dialysis as risk to their kidney function was no longer relevant, currently pregnant or unable to provide informed consent.

Clinical care teams recruited women face-to-face or by telephone. A study invitation, information sheet, data protection document and study hyperlink was sent to their e-mail address. Paper versions were also offered to reduce bias. Informed consent was obtained before any data were collected.

Clinical characteristics recorded outside of pregnancy within the last 2 years including most recent estimated glomerular filtration rate (eGFR) using Chronic Kidney Disease Epidemiology Collaboration equation (CKD-EPI) [20] without ethnicity adjustment, primary CKD diagnosis, number of inpatient admissions in the past 5 years, dialysis and transplant history, proteinuria, blood pressure and antihypertensive history were extracted from local databases by clinical care teams. Single measurements of eGFR were used as women were under the care of their local renal team and had an established CKD diagnosis. Clinically relevant cut points were used to identify proteinuria as participating sites recorded either Albumin: creatinine ratio (ACR) or Protein: creatinine ratio (PCR). The cut points were: ACR > 70 mg/mmol or PCR > 100 mg/mmol. Variables are described in Supplementary Table 1.

### Measures

Demographic and social information, and perspectives of pregnancy were collected via a self-report questionnaire. Perception of pregnancy risk was assessed by a modified version of the Perception of Pregnancy Risk Questionnaire (PPRQ) [21]. Results are reported as an overall mean score, and two subscale scores: Risk to Self and Risk to Baby. The greater the score, the greater the perceived pregnancy risk. Modifications of the PPRQ for the purposes of this study included alteration of tense to enable completion outside of pregnancy and adaptation to include kidney disease. Content validity of the modified PPRQ was assessed. Summarised changes to the PPRQ and content validity results are presented in supplementary file 1 and supplementary table 2. Pregnancy intention was measured by the Desire to Avoid Pregnancy Scale (DAP) [22]. For application in this study a high score indicated stronger pregnancy intention and a lower score indicated stronger pregnancy avoidance.

The following psychological attributes were also measured; depression and anxiety using the Patient Health Questionnaire for Depression and Anxiety (PHQ-4) [23], illness perceptions using Brief Illness Perceptions Questionnaire (B-IPQ) [24], social support using Multidimensional Scale of Perceived Social Support (MSPSS) [25], quality of life using the Kidney Disease Quality of Life Instrument Short Form (KDQOL-SF version 1.3)[26] with sub-scales Physical Component Score (PCS), Mental Component Score (MCS) and Kidney Disease Component Score (KDCS) and lastly COVID-19 risk perception was established and controlled for in the analyses using the COVID-19 Risk Perception Score [27]. These measures are described further in Supplementary Table 1.

### Statistical analysis

A priori power analysis was conducted to determine the necessary sample size. Standardised measures were scored following authors’ instructions and summarised alongside baseline and demographic data. Groupwise comparisons between CKD stage and the summarised variables were made (Supplementary Table 3). Correlation between PPRQ subscales was assessed using Pearson *r* and latent factors modelled. Reliability of the PPRQ scale measure and one summary KDQOL score was determined by Cronbach’s alpha scores ($\alpha$). The KDQOL subscale was chosen based on the Pearson *r* correlation between the three summary scores and other psychological attributes measured, the subscale score with weakest association with the other attributes was used. Significance was set at *P* ≤ 0.05, using the set alpha (α) value of 0.05.

Missing data were described and imputed using multiple imputation with 20 imputations. For sensitivity, the analyses were repeated using listwise deletion and results were compared (Supplementary materials: Tables 4, 5, 6, 7, 8).

There were two outcome measures; perception of pregnancy risk and pregnancy intention. Initial independent sample t-tests were performed examining perception of pregnancy risk and pregnancy intention between women with different severities of CKD (stage 1–2 compared with CKD stages 3–5) and transplant recipients compared with non-transplant recipients.

Univariate linear regression was used to explore the association between variables and perceived pregnancy risk and pregnancy intention. Hierarchical linear regression was used for variable selection for separately assessing association between perception of pregnancy risk and pregnancy intention. The first block adjusted for demographic characteristics that had significant univariate associations, and clinically relevant characteristics (eGFR, history of previous dialysis, transplant, clinically relevant proteinuria, and chronic hypertension), attendance to pre-pregnancy counselling was included as a confounding factor. The second block adjusted for psychological attributes that were significantly associated in the univariate regression. The variance explained (*R*^2^) was reported in all models, and multivariate Wald test was performed to evaluate the difference between nested models.

Pearson *r* correlation was performed to assess whether there is an association between perception of pregnancy risk and pregnancy intention with the overall cohort and subgroups with varying severities of CKD: stage 1–2, stages 3–5, non-transplant recipients and transplant recipients.

## Results

A total of 716 women were contacted to participate in the study, there was a 44% response rate with 315 women included in the analysis, the majority of whom were recruited from London clinics (251, 80%) (Supplementary Table 9). The mean age was 35 years (SD 7.1) (Table 1) and the median pre-pregnancy eGFR was 64 (IQR 56) mL/min/1.73m^2^. Ninety-four (30%) were renal transplant recipients, 53 (56%) of whom were pre-emptive. Over half had at least one pregnancy (186, 59%) and 46 (14.6%) were currently trying to conceive at time of participating. Most women reported pregnancy being important or very important to themselves (234; 74.3%) and to their family (211; 67%).

**Table 1:** Summary of demographic, clinical and psychological variables

Variable		Overall (*N* = 315)
Age (years) (mean (SD))		35.0 (7.1)
Missing		0
Ethnicity (%)		
Asian		36 (11.4)
Black		47 (14.9)
White		203 (64.4)
Mixed		13 (4.1)
Other		15 (4.8)
Missing		1 (0.3)
Education (%)		
No formal education		4 (1.3)
GCSE (full time education to age 16)		62 (19.7)
A Level (full time education to age 18)		60 (19.0)
Undergraduate degree		102 (32.4)
Postgraduate degree		87 (27.6)
Online completion (%)		315 (100%)
Employment (%)		
Full-time		152 (48.3)
Part-time		78 (24.8)
Home maker		26 (8.3)
Retired		1 (0.3)
Unemployed		30 (9.5)
Student		13 (4.1)
Other		14 (4.4)
Missing		1 (0.3)
Living arrangement (%)		
Living with partner		203 (64.4)
Living with relatives/friends		63 (20.0)
Living alone		47 (14.9)
Missing		2 (0.6)
Socio-economic status^a^ (median [IQR])		5.0 [3.0,7.0]
Missing		12
Religion (%)		
No religion		142 (45.1)
Christian		131 (41.6)
Buddhist		1 (0.3)
Hindu		11 (3.5)
Jewish		2 (0.6)
Muslim		17 (5.4)
Sikh		2 (0.6)
Other		8 (2.5)
Missing		1 (0.3)
Actively practising religion (%)	Yes	89 (28.3)
Missing		2 (0.6)
**Self-reported pregnancy preferences**		
Pregnancy importance (%)		
Unimportant		31 (9.8)
Slightly important		14 (4.4)
Moderately important		36 (11.4)
Important		75 (23.8)
Very important		159 (50.5)
Missing		0
Importance of pregnancy to family (%)		
Unimportant		38 (12.1)
Slightly important		17 (5.4)
Moderately important		49 (15.6)
Important		99 (31.4)
Very important		112 (35.6)
Missing		0
Attended pre pregnancy counselling (%)	Yes	108 (34.3)
Missing		1 (0.3)
Currently trying for pregnancy (%)	Yes	46 (14.6)
Missing		2 (0.6)
Previous pregnancy^b^	Yes	186 (59.0)
Missing		3 (1.0)
Perceived severity of kidney disease (0–100) (median [IQR])		50 [25, 70]
Missing		5
**Clinical summary**		
Cause of CKD (%)		
Glomerulonephritis		64 (20.3)
Chronic progressive nephropathy/ vesicoureteral reflux		51 (16.2)
Autosomal dominant polycystic kidney disease		47 (14.9)
Diabetic nephropathy		24 (7.6)
Congenital/inherited		30 (9.5)
Transplant		9 (2.9)
Other		38 (12.1)
Systemic lupus erythematosus		35 (11.1)
Unknown		6 (1.9)
Missing		11 (3.5)
eGFR^c^ (ml/min/1.73m^2^) (median [IQR])		64 [37.0, 93.0]
Missing		4
No. of inpatient admissions in the past five years (median [IQR])		1.00 [0.00, 3.00]
Previous dialysis (%)	Yes	50 (15.9)
Missing		3 (1.0)
Previous transplant (%)		
No		220 (69.8)
Yes–pre-emptive		74 (23.6)
Yes–not pre-emptive		20 (6.3)
Missing		1 (0.3)
Chronic hypertension^d^ (%)	Yes	167 (53.0)
Missing		23 (7.3)
Clinically relevant proteinuria^e^ (%)	Yes	56 (17.8)
Missing		105 (33.3)

	Mean (SD)	Median (IQR)	Min and max	Skewness	Kurtosis	Missing	Cronbach’s $\alpha$
**Psychological summary**							
Perceived pregnancy risk^f^							
Overall	46 (23)	46 (34.2)	0–100	0.2	− 0.5	19 (6%)	0.92
Risk to baby	44 (25)	43 (36.6)	0–100	0.3	− 0.6	17 (5.4%)	0.91
Risk to mother	48 (23)	48 (32.8)	0–100	0.1	− 0.5	9 (2.9%)	0.81
COVID-19 risk perception^g^	5 (1)	5 (1.3)	1–7	− 0.4	0.5	2 (0.6%)	0.78
Pregnancy intention^h^	2 (1)	2 (1.8)	0–4	− 0.1	− 1.0	6 (1.9%)	0.96
Distress^i^	3 (3)	2 (4)	0–12	1.2	0.8	4 (1.3%)	0.90
Perceived social support^j^							
Overall perceived social support	6 (1)	6 (1.4)	1–7	− 1.6	3.2	7 (2.2%)	0.95
From significant other	6 (1)	6 (1.2)	1–7	− 1.8	3.1	1(0.3%)	0.96
From friends	6 (1)	6 (2.0)	1–7	− 1.6	2.6	5 (1.6%)	0.96
From family	6 (1)	6 (1.6)	1–7	− 1.6	2.3	3 (1%)	0.94
Illness perceptions^k^	47 (13)	48 (16)	0–78	− 0.4	0.4	3 (1%)	0.71
Quality of Life^l^							
Physical component summary	66 (25)	76 (36.7)	2–100	− 0.9	− 0.4	17 (5.4%)	0.95
Mental component summary	63 (24)	71 (40.1)	2–100	− 0.7	− 0.7	32 (10.2%)	0.91
Kidney disease component summary	76 (15)	79 (19.4)	32–97	− 1.0	0.5	123 (39%)	0.94

*eGFR* estimated glomerular filtration rate; *CKD* chronic kidney disease

^a^Socioeconomic status is measured using the Index of Multiple Deprivation (IMD). The IMD measures relative deprivation across each small area in England in deciles, where 1 represents the most deprived 10% to 10 which represents the least deprived 10 percent. Deprivation is measures across seven domains; income, employment, education, health, crime, barriers to housing and services, and living environment

^b^Self-reported pregnancy history

^c^Calculated using Chronic Kidney Disease Epidemiology Collaboration 2009 equation without ethnicity adjustment

^d^History of antihypertensives and/or > 140 mmHg/ > 90 mmHg

^e^Albumin: creatinine ratio > 70 mg/mmol or protein: creatinine ratio > 100 mg/mmol

^f^Measured using a modified version of Perception of Pregnancy Risk Questionnaire, increased score indicates greater perceived risk (0–100)

^g^Measured using COVID-19 Risk Perception Score, increased score indicates greater perceived COVID risk (1—7)

^h^Measured using Desire to Avoid Pregnancy Scale, increased score indicates greater pregnancy intention (0–4)

^i^Measured using Patient Health Questionnaire 4 Item Scale, high score indicates greater anxiety and depression (0–12)

^j^Measured using Multidimensional Scale of Perceived Social Support, increased score indicates greater social support (1–7)

^k^Measured using Brief Illness Perceptions Questionnaire, increased score indicates more negative illness beliefs (0–78)

^l^Measured using Kidney Disease Quality of Life Scale, increased score indicates greater quality of life (0–100)

The majority of women had not attended pre-pregnancy counselling (206, 65.4%). Those who had attended had more advanced CKD (median pre-pregnancy eGFR 54 mL/min/1.73m^2^ versus 68 mL/min/1.73m^2^, *p* = 0.007), perceived greater pregnancy risk (mean overall perceived pregnancy risk score 51.7 versus 42.5, *p* = 0.001) and had higher education (71.3% versus 54.4% university graduates, *p* = 0.001) (Supplementary Table 10).

The subscales of perception of pregnancy risk questionnaire (PPRQ) were strongly correlated (*r* = 0.81). When latent factors were modelled correlation increased (*r* = 0.90), thus the overall perceived pregnancy risk scale was used in the analysis. Correlations between KDQOL summary scores and the other psychological constructs confirmed that the MCS (*r* = − 0.75, 95% CI − 79 to − 0.69) and KDCS had strong negative correlations with distress (*r* − 0.66, 95% CI − 0.73 to − 0.57, *p* < 0.00). The PCS did not correlate with the psychological attributes measured (*r *= $\le$ 0.50) so the PCS was used as the QoL indicator in regression analyses to avoid collinearity with other variables (Supplementary Table 11).

### Perception of pregnancy risk

There was a significant difference in the overall perceived pregnancy risk of women with CKD stages one to two (PPRQ mean 38.7, SD = 22.9) and stages three to five (PPRQ mean 53.2, SD = 23.1); *t*(280) = − 5.6, 95% CI − 19.6 to − 9.4, *p* < 0.001. There was also a difference in perceived pregnancy risk between women who had received a kidney transplant (PPRQ mean 55.5, SD = 22.5) compared to those who had not (PPRQ mean 41.3, SD = 23.5): *t*(295) = -5.2, 95% CI − 19.7 to − 8.7, *p* < 0.001.

Univariate analysis identified older age, pre-pregnancy counselling attendance, greater perceived severity of CKD, previous dialysis, kidney transplantation, clinically relevant proteinuria, and chronic hypertension as significantly associated with higher perceived pregnancy risk. Preserved kidney function, greater perceived quality of life, and being employed were significantly associated with a lower perception of pregnancy risk. Women who perceived their CKD with more negative beliefs, experienced greater anxiety and depression (distress), or reported a perceived greater risk of COVID-19 had increased perceived pregnancy risk (Table 2).

**Table 2:** Assessing the univariate relationships between individual variables with perception of pregnancy risk and pregnancy intention as dependent variables

Variable	Perception of pregnancy risk		Pregnancy intention	
	Coefficients	*p*	Coefficients	*p*
Age	0.5 (0.1–0.8)	0.017	− 0.01 (− 0.03 to 0.01)	0.22
Ethnicity–Asian	7.4 (− 1.2 to 16.0)	0.091	0.9 (0.5–1.3)	< 0.001
Ethnicity–Black	1.5 (− 6.0 to 8.9)	0.70	0.4 (0.1–0.7)	0.026
Ethnicity–Other	3.8 (− 5.7 to 13.4)	0.43	0.2 (− 0.3 to 0.6)	0.48
Education–GCSE	− 7.9 (− 31.4 to 15.7)	0.51	− 0.2 (− 1.4 to 0.9)	0.73
Education – A level	− 14.5 (− 38.0 to 9.0)	0.22	− 0.02 (− 1.2 to 1.1)	0.97
Education–Undergraduate	− 18.4 (− 41.6 to 4.8)	0.12	− 0.2 (− 1.3 to 1.0)	0.79
Education – Postgraduate	− 16.1 (− 39.3 to 7.2)	0.18	− 0.2 (− 1.3 to 1.0)	0.78
Employed – Yes	− 7.4 (− 13.3 to 1.5)	0.015	0.1 (− 0.2 to 0.4)	0.62
Living arrangement – relatives/friends	− 1.7 (− 8.6 to 5.1)	0.62	− 0.3 (− 0.6 to 0.0)	0.072
Living arrangement – alone	0.6 (− 6.9 to 8.1)	0.87	− 0.3 (− 0.7 to 0.1)	0.089
Socio-economic status^a^	− 0.7 (− 1.8 to 0.3)	0.18	− 0.04 (− 0.1 to 0.1)	0.085
Religious–yes	1.8 (− 3.5 to 7.1)	0.50	0.5 (0.3–0.8)	< 0.001
Importance of pregnancy	0.5 (− 1.5 to 2.5)	0.64	0.4 (0.3–0.5)	< 0.001
Importance of pregnancy to family	0.1 (− 1.9 to 2.1)	0.92	0.3 (0.2–0.4)	< 0.001
Attended PPC	8.9 (3.4–14.4)	0.002	0.6 (0.3–0.8)	< 0.001
Pregnancy history	1.3 (− 0.2 to 2.8)	0.08	− 0.04 (− 0.1 to 0.03)	0.27
Children	0.7 (− 2.2 to 3.5)	0.64	− 0.2 (− 0.4 to − 0.1)	0.001
Perceived CKD severity	0.4 (0.3–0.5)	< 0.001	0.002 (0.006 to 0.003)	0.51
**Clinical characteristics**				
Previous Dialysis–yes	11.1 (4.0–18.1)	0.002	0.1 (− 0.3 to 0.4)	0.65
Previous Transplant–yes	14.2 (8.7–19.7)	< 0.001	0.1 (− 0.2 to 0.3)	0.70
eGFR^b^	− 0.2 (− 0.3 to − 0.2)	< 0.001	0.0001 (− 0.004 to 0.004)	0.97
Clinically relevant proteinuria^c^	9.1 (1.8–16.4)	0.016	− 0.1 (− 0.4 to 0.3)	0.64
Chronic hypertension^d^	7.9 (2.4–13.4)	0.005	0.1 (− 0.1 to 0.4)	0.29
**Psychological characteristics**				
COVID – risk perception^e^	5.2 (2.7–7.8)	< 0.001	− 0.2 (− 0.4 to − 0.1)	< 0.001
Pregnancy intention^f^	− 0.04 (− 2.5 to 2.4)	0.97	–	-
Pregnancy risk perception^h^	–	–	0.0001 (− 0.01 to 0.01)	0.97
Distress^i^	1.3 (0.5–2.2)	0.002	− 0.1 (− 0.09 to − 0.01)	0.009
Social support^j^	− 0.8 (− 3.1 to 1.5)	0.52	0.1 (− 0.03 to 0.2)	0.15
Illness perceptions^k^	0.8 (0.6–1.0)	< 0.001	− 0.004 (− 0.01 to 0.005)	0.39
Quality of life^11^ – physical	− 0.2 (− 0.3 to − 0.04)	0.006	0.01 (0.001–0.01)	0.013

Reported to one significant figure after decimal

*IMD* index of multiple deprivation; *PPC* pre-pregnancy counselling; *eGFR* estimated glomerular filtration rate; *CKD* chronic kidney disease. reference categories: ethnicity = white ethnicity, education = no formal education, living arrangement = living with partner

^a^Socioeconomic status is measured using the Index of Multiple Deprivation

^b^eGFRcalculated using Chronic Kidney Disease Epidemiology Collaboration 2009 equation without ethnicity adjustment

^c^Albumin:creatinine ratio > 70 mg/mmol or Protein: creatinine ratio > 100 mg/mmol

^d^History of antihypertensives and/or > 140 mmHg/ > 90 mmHg

^e^Measured using COVID-19 Risk Perception Score

^f^Measured using Desire to Avoid Pregnancy Scale

^g^Measured using a modified version of Perception of Pregnancy Risk Questionnaire

^h^Measured using Patient Health Questionnaire 4 Item Scale

^i^Measured using Multidimensional Scale of Perceived Social Support

^j^Measured using Brief Illness Perceptions Questionnaire

^k^Measured using Kidney Disease Quality of Life Scale – physical component summary

After inclusion of demographic data, psychological attributes and relevant clinical characteristics in the model, pre-pregnancy counselling attendance, greater perceived severity of CKD, more negative illness beliefs and greater COVID-19 risk perception remained significantly associated with greater perceived pregnancy risk (Table 3). There was no association between clinical characteristics and perceived pregnancy risk. Inclusion of psychological variables within the model improved explained variance from 21% (*R*^2^ = 0.21, 95% CI 0.13–0.30) to 33% (*R*^2^ = 0.33, 95% CI 0.24–0.42). The Wald test was significant (*p* < 0.001) indicating that psychological attributes significantly contribute to the model of perceived pregnancy risk.

**Table 3:** Adjusted linear regression models investigating the association with perception of pregnancy risk

	Perception of pregnancy risk model			
	Model 1		Model 2	
	Coefficients	*p*	Coefficients	*p*
Intercept	46.5 (30.4–62.5)	< 0.001	− 7.9 (-33.5 to 17.6)	0.54
**Demographic factors**				
Age	0.1 (− 0.2 to 0.5)	0.43	0.1 (− 0.3 to 0.4)	0.68
Employed–Yes	− 6.1 (− 11.6 to -0.7)	0.028	− 3.7 (− 9.1 to 1.7)	0.17
PPC attended	6.8 (1.6–12.0)	0.011	6.5 (1.5–11.4)	0.011
**Clinical characteristics**				
eGFR^a^	− 0.2 (− 0.2 to − 0.1)	< 0.001	− 0.03 (− 0.1 to 0.1)	0.49
Previous dialysis–yes	3.5 (− 4.2 to 11.2)	0.37	1.5 (-5.7 to 8.8)	0.68
Previous transplant–yes	7.8 (1.4–14.3)	0.017	4.7 (− 1.5 to 10.9)	0.14
Clinically relevant proteinuria^b^–yes	3.9 (− 3.9 to 11.6)	0.33	5.0 (− 2.9 to 12.9)	0.21
Chronic hypertension^c^–yes	4.2 (− 1.2 to 9.6)	0.13	2.0 (− 3.3 to 7.3)	0.47
**Psychological attributes**				
COVID risk perception^d^	–	–	2.7 (0.3–5.2)	0.031
Distress^e^	–	–	0.7 (− 0.2 to 1.6)	0.14
Illness perceptions^f^	–	–	0.4 (0.2–0.7)	0.001
Quality of life^g^–PCS	–	–	0.1 (− 0.03 to 0.2)	0.13
Perceived CKD severity	–	–	0.1 (0.04–0.3)	0.010
**Model summary**				
*R*^2^	0.21 (0.13–0.30)		0.33 (0.24–0.42)	
Δ*R*^2^	0.19 (0.12–0.28)		0.30 (0.21–0.39)	

Reported to one significant figure after decimal. Model 1 adjusts for demographic and clinical factors only. Model 2 adjusts for demographic, clinical and psychological factors

*PPC* pre-pregnancy counselling; *eGFR* estimated glomerular filtration rate; *CKD* chronic kidney disease; *R*^*2*^ *R* squared; *ΔR*^*2*^ Adjusted R square

^a^eGFRcalculated using Chronic Kidney Disease Epidemiology Collaboration 2009 equation without ethnicity adjustment

^b^Albumin:creatinine ratio > 70 mg/mmol or Protein: creatinine ratio > 100 mg/mmol

^c^History of antihypertensives and/or > 140 mmHg/ > 90 mmHg

^d^Measured using COVID-19 Risk Perception Score

^e^Measured using Patient Health Questionnaire 4 Item Scale

^f^Measured using Brief Illness Perceptions Questionnaire

^g^Measured using Kidney Disease Quality of Life Scale physical component summary

### Pregnancy intention

There was no significant difference in pregnancy intentions between women with CKD stages one to two (pregnancy intention mean score = 2, SD = 1.1) and stages three to five (pregnancy intention mean score = 2.1, SD = 1.2); *t*(303) = -0.5, 95% CI -0.3 to 0.2, *p* = 0.647. There was no significant difference in pregnancy intentions between women who had received a kidney transplant (pregnancy intention mean score = 2.1, SD = 1.2) compared to those who had not (pregnancy intention mean score = 2.0, SD = 1.1): *t*(307) =  − 0.4, 95% CI − 0.33 to 0.22,* p* = 0.697.

In the univariate analyses religious identity, Black or Asian ethnicity, attendance at pre-pregnancy counselling, regarding pregnancy as important to themselves and their families, and greater quality of life were all measurably associated with greater pregnancy intention. Conversely, greater perceived COVID-19 risk, greater distress, and increased parity were associated with avoidance of pregnancy. Clinical characteristics were not associated with pregnancy intention (Table 2).

In the multivariable model, pregnancy counselling attendance, religious identity and regarding pregnancy with greater importance were significantly associated with greater pregnancy intention (Table 4). An increase in number of children, and greater perceived risk of COVID-19 were associated with avoidance of pregnancy. No association was identified between clinical characteristics and pregnancy intention. Inclusion of psychological variables within the model improved explained variance from 33% (*R*^2^ = 0.33, 95% CI 0.25–0.42) to 36% (*R*^2^ = 0.36, 95% CI 0.28–0.45) with significant Wald test (*p* = 0.005) indicating that psychological attributes contribute to the model explaining pregnancy intention.

**Table 4:** Adjusted linear regression models investigating associations with pregnancy intention as outcome

	Variable	Model 1		Model 2	
		Coefficients	*p*	Coefficients	*p*
Demographic factors	Intercept	0.4 (− 0.1 to 0.9)	0.11	0.9 (0.1–1.7)	0.035
	Ethnicity–Asian	0.3 (− 0.1 to 0.7)	0.13	0.3 (− 0.1 to 0.7)	0.13
	Ethnicity–Black	0.02 (− 0.3 to 0.4)	0.89	− 0.003 (− 0.3 to 0.3)	0.99
	Ethnicity–Other	− 0.01 (− 0.4 to 0.4)	0.94	0.1 (− 0.3 to 0.5)	0.69
	Religion–yes	0.3 (0.05–0.5)	0.019	0.3 (0.03–0.5)	0.028
	Importance of pregnancy	0.4 (0.2 to 0.5)	< 0.001	0.4 (0.2–0.5)	< 0.001
	Importance of pregnancy to family	0.01 (− 0.1 to 0.1)	0.87	0.02 (− 0.1 to 0.2)	0.76
	Attended PPC	0.3 (0.05–0.5)	0.019	0.3 (0.01–0.5)	0.041
	Children	− 0.31 (− 0.4 to − 0.2)	< 0.001	− 0.3 (− 0.4 to − 0.2)	< 0.001
Clinical characteristics	eGFR^a^	0.0002 (− 0.003 to 0.004)	0.92	0.0005 (− 0.004 to 0.003)	0.79
	Previous dialysis–yes	0.3 (− 0.1 to 0.6)	0.12	0.3 (− 0.1 to 0.6)	0.096
	Previous transplant–yes	− 0.02 (− 0.3 to 0.3)	0.87	0.005 (− 0.3 to 0.3)	0.97
	Clinically relevant proteinuria^b^–yes	− 0.2 (− 0.5 to 0.1)	0.11	− 0.2 (− 0.5 to 0.1)	0.11
	Chronic hypertension^c^–yes	0.2 (− 0.1 to 0.4)	0.16	0.2 (− 0.001 to 0.5)	0.051
Psychological attributes	COVID risk perception^d^	–	–	− 0.1 (− 0.2 to -0.01)	0.029
	Quality of life–physical^e^	–	–	0.003 (− 0.002 to 0.01)	0.28
	Distress^f^	–	–	− 0.02 (− 0.1 to 0.02)	0.25
Model summary	R^2^	0.33 (0.25–0.42)		0.36 (0.28 to 0.45)	
	ΔR^2^	0.3 (0.22–0.39)		0.33 (0.24 to 0.41)	

Reported to one significant figure after decimal. Model 1 adjusts for demographic and clinical factors only. Model 2 adjusts for demographic, clinical and psychological factors

*PPC* pre-pregnancy counselling; *eGFR* estimated glomerular filtration rate; CKD chronic kidney disease; *R*^*2*^  *R* squared; *ΔR*^*2*^ adjusted *R* square

^a^eGFR calculated using Chronic Kidney Disease Epidemiology Collaboration 2009 equation without ethnicity adjustment

^b^Albumin: creatinine ratio > 70 mg/mmol or Protein: creatinine ratio > 100 mg/mmol

^c^History of antihypertensives and/or > 140 mmHg/ > 90 mmHg

^d^Measured using COVID-19 Risk Perception Score

^e^Measured using Kidney Disease Quality of Life Scale physical component summary

^f^Measured using Patient Health Questionnaire 4 Item Scale

There was no association between perception of pregnancy risk and pregnancy intention (*r* = − 0.002, 95% CI − 0.12 to 0.11, *p* = 0.97). No association was consistent in the subgroup analyses amongst: CKD stages 1 to 2 (*r* = 0.09, 95% CI − 0.08 to 0.25, *p* = 0.30), CKD stages 3 to 5 (*r* = − 0.08, 95% CI -0.24 to 0.09, *p* = 0.35), kidney transplant recipients (*r* = − 0.1, 95% CI − 0.3 to 0.12, *p* = 0.38) and non-kidney transplant recipients (*r* = 0.04, 95% CI − 0.1 to 0.18, *p* = 0.57).

## Discussion

Clinical risk factors for adverse pregnancy outcomes including eGFR, hypertension and proteinuria did not affect perception of pregnancy risk or future pregnancy intention in women with CKD in the adjusted analyses. Severity of CKD and previous transplant history had no association with pregnancy intention. Women’s own perception of CKD severity was associated with increased perceived pregnancy risk, but this did not influence women’s pregnancy intentions, thus highlighting the priority of pregnancy for many women. Women who have previously attended pre-pregnancy counselling have greater perceived pregnancy risk and pregnancy intention.

Women with CKD have greater pregnancy risk perception scores in comparison to other tested cohorts. Previous risk perception scores in uncomplicated pregnancies were 24.0 (SD 14.5) [21], substantially lower than the overall scores reported in this study (PPRQ overall: 46, SD 23). Women with reduced kidney function (CKD stage 3–5) reported to perceive greater pregnancy risk in comparison to women with CKD stages 1–2. Overall, we observed that measured kidney function has no association with either perceived pregnancy risk or pregnancy intention, and women’s own perception of their CKD severity had a stronger impact upon their perceived pregnancy risk than measured kidney function. This is in agreement with previous studies that report differences between women’s perceived risk and that of a healthcare professional or an objective risk assessment [13, 14].

Qualitative studies report the decisional conflict between balancing pregnancy desire and pregnancy risk related to CKD [10]. This study demonstrates that for many women with CKD pregnancy desire outweighs perceived risk as perception of pregnancy risk did not impact pregnancy intention across the cohort nor in subgroups with varying severities and transplant history. This finding is not aligned with psychological theories that propose behaviour intention can be explained and predicted by perceived risk along with other psychological constructs [15, 16]. The psychological cost that may be incurred by avoiding pregnancy should also be acknowledged [15]. For many women, pregnancy is valued as a life ambition and thus the psychological cost of avoiding pregnancy may exceed potential risk.

Similar to other reports, attendance to pre-pregnancy counselling was low [10], but women who had attended pre-pregnancy counselling reported greater risk perception, which may be due to being more informed. Alternatively, women who attend pre-pregnancy counselling may have pre-existing greater perceived pregnancy risk, which underlies their motivation to attend. Assessment of risk perception before and after pre-pregnancy counselling would provide further insight. Pre-pregnancy counselling was attended by women with more clinical risk factors, which likely reflects referral pathways for women with increased clinical risk. However, pre-pregnancy counselling was also attended by women with higher levels of educational achievement, perhaps highlighting inequality of access. Although the lack of an association between clinical risk factors and a woman’s perception of pregnancy risk presented here may be due to the importance of pregnancy to women regardless of clinical status, it may also demonstrate limited awareness of actual risk that could be improved with pre-pregnancy counselling. All women with CKD should therefore be offered pre-pregnancy counselling, with attendance facilitated and language barriers addressed to reduce inequality.

The study findings need to be interpreted considering its limitations. Factors that may influence women’s perceptions of pregnancy risk have not been adjusted for in the analysis due to restricted access to data or unreliable reporting. This includes individual complexities of uncontrolled disease and medication history which may influence perceptions of pregnancy risk due to possible teratogenic risks [28]. In addition, the availability heuristic proposes that women’s previous experiences of pregnancy, including complications such as pre-eclampsia and pre-term deliveries, as well as what stage they are at in their pregnancy journey, may also be used to formulate their perception of pregnancy risk and pregnancy intention [29]. It is important to consider the possible bias underpinning attendance to pre-pregnancy counselling clinics and how this attendance may subsequently influence pregnancy risk perception.

The PPRQ was modified for this research and whilst the content validity of the instrument was assessed, further psychometric validation assessment is recommended. Secondly, women participated during the COVID-19 pandemic and so the data collected was a snapshot at a time where pregnancy intention and risk perception may have changed. Furthermore, the majority of the cohort had an undergraduate degree or above, in comparison to 40.6 percent in the United Kingdom having post-secondary school educational qualification [30], thus is unlikely to be representative of all women with CKD. Digital exclusion may be contributory, although postal questionnaires were offered. In addition, women were recruited from metropolitan areas within the United Kingdom with access to pre-pregnancy counselling, which would differ for women from rural areas and may either increase or reduce their perceptions of pregnancy risk.

It is important to note a large proportion of the cohort were White women. It is possible that women from different ethnicities may have different perceptions of pregnancy risk, for instance people of African or Afro-Caribbean ancestry are at greater risk of CKD progression [31], adverse pregnancy outcomes and mortality [32] which may heighten their perception of pregnancy risk. Lastly, the findings cannot be generalised to a non-English speaking population.

This is the first study that has investigated perceptions of pregnancy risk and pregnancy intentions in women with CKD and has highlighted that pregnancy desire may be unwavering regardless of perceived or objective pregnancy risk. Pregnancy is important to women with CKD, but frequently women are unaware of their risk of adverse pregnancy outcomes. The clinical implication of these findings is that there needs to be greater efforts to support physical and psychological optimisation prior to pregnancy for women with CKD. This includes greater emphasis for women with CKD to receive pre-pregnancy counselling as recommended by the mentioned guidelines to facilitate informed decision making [4, 11]. Pre-pregnancy counselling should include the successful communication of clinical risk including adverse pregnancy and renal outcomes. However, shared-decision making regarding pregnancy requires an understanding of perceived risk, and the importance of pregnancy.

## Supplementary Information

Below is the link to the electronic supplementary material.

Supplementary file1 (DOCX 150 KB)

## Data Availability

The datasets generated during and/or analysed during the current study are available from the corresponding author on reasonable request.

## References

[1] Piccoli GB, Attini R, Vasario E (2010). Pregnancy and chronic kidney disease: a challenge in all CKD stages. Clin J Am Soc Nephrol.

[2] MacLaughlin HL, Hall WL, Sanders TA, Macdougall IC (2015). Risk for chronic kidney disease increases with obesity: health survey for England 2010. Public Health Nutr.

[3] Wu B, Bell K, Stanford A (2016). Understanding CKD among patients with T2DM: prevalence, temporal trends, and treatment patterns—NHANES 2007–2012. BMJ Open Diabetes Res Care.

[4] Wiles K, Chappell L, Clark K (2019). Clinical practice guideline on pregnancy and renal disease. BMC Nephrol.

[5] Piccoli GB, Cabiddu G, Attini R (2015). Risk of adverse pregnancy outcomes in women with CKD. J Am Soc Nephrol.

[6] Wiles K, Webster P, Seed P (2020). The impact of chronic kidney disease Stages 3–5 on pregnancy outcomes. Nephrol Dial Transpl.

[7] Harel Z, Park A, McArthur E (2020). Prepregnancy renal function and risk of preterm birth and related outcomes. CMAJ.

[8] al Khalaf S, O’Reilly É, McCarthy F (2021). Pregnancy outcomes in women with chronic kidney disease and chronic hypertension: a National cohort study. Am J Obstet Gynecol.

[9] Hall M, Lightstone L (2018). Prepregnancy counseling and risk assessment. Renal disease in pregnancy.

[10] Jesudason S, Tong A (2019). The patient experience of kidney disease and pregnancy. Best Pract Res Clin Obstet Gynaecol.

[11] Centre for Maternal and Child Enquiries (2011) Saving mothers’ lives: reviewing maternal deaths to make motherhood safer: 2006–08. In: The Eighth Report on Confidential Enquiries into Maternal Deaths in the United Kingdom 10.1111/j.1471-0528.2010.02847.x 21356004

[12] Lee S (2014). Risk perception in women with high-risk pregnancies. Br J Midwifery.

[13] Heaman M, Beaton A, Gupton A, Sloan J (1992). A comparison of childbirth expectations in high-risk and low-risk pregnant women. Clin Nurs Res.

[14] Gray B (2006). Hospitalization history and differences in self-rated pregnancy risk. West J Nurs Res.

[15] Janz N, Becker M (1984). The health belief model. A decade later. Health Educ Q.

[16] Rogers R (1975). A protection motivation theory of fear appeals and attitude change. J Psychol.

[17] Widnes SF, Schjøtt J, Granas AG (2012). Risk perception and medicines information needs in pregnant women with epilepsy: a qualitative study. Seizure.

[18] Kim C, McEwen L, Piette J (2007). Risk perception for diabetes among women with histories of gestational diabetes mellitus. Diabetes Care.

[19] Kidney Disease: Improving Global Outcomes (2012). KDIGO 2012 clinical practice guideline for evaluation and management of CKD. Kidney Int Suppl.

[20] Levey A, Stevens L, Schmid C (2009). A new equation to estimate glomerular filtration rate. Ann Intern Med.

[21] Heaman M, Gupton A (2009). Psychometric testing of the perception of pregnancy risk questionnaire. Res Nurs Health.

[22] Rocca CH, Ralph LJ, Wilson M (2019). Psychometric evaluation of an instrument to measure prospective pregnancy preferences. Med Care.

[23] Kroenke K, Spitzer RL, Williams J, Lowe B (2009). An ultra-brief screening scale for anxiety and depression: the PHQ-4. Psychosomatics.

[24] Broadbent E, Petrie KJ, Main J, Weinman J (2006). The brief illness perception questionnaire. J Psychosom Res.

[25] Zimet G, Dahlem N, Zimet S, Farley G (1988). The multidimensional scale of perceived social support. J Pers Assess.

[26] Hays R, Kallich J, Mapes D (1994). Development of the kidney disease quality of life KDQOL instrument. Qual Life Res.

[27] Dryhurst S, Schneider CR, Kerr J (2020). Risk perceptions of COVID-19 around the world. J Risk Res.

[28] Widnes S, Schjøtt J (2017). Risk perception regarding drug use in pregnancy. Am J Obstet Gynecol.

[29] Tversky A, Kahneman D (1973). Availability: a heuristic for judging frequency and probability. Cogn Psychol.

[30] Eurostat (2022) Population by educational attainment level, sex and age (%) main indicators. https://appsso.eurostat.ec.europa.eu/nui/submitViewTableAction.do. Accessed 5 May 2022

[31] National Kidney Foundation (2002). K/DOQI clinical practice guidelines for chronic kidney disease: evaluation, classification, and stratification. Am J Kidney Dis.

[32] MacDorman MF, Thoma M, Declcerq E, Howell EA (2021). Racial and ethnic disparities in maternal mortality in the United States using enhanced vital records, 2016–2017. Am J Public Health.

