# Supplementary material for: Exploring biopsychosocial correlates of pregnancy risk and pregnancy intention in women with chronic kidney disease
Source: J Nephrol. 2023 Mar 27;36(5):1361–72. doi: 10.1007/s40620-023-01610-2 (PMC10041500; doi:10.1007/s40620-023-01610-2)
Supplement: Supplementary file 1 — Supplementary file1 (DOCX 150 KB) [file 40620_2023_1610_MOESM1_ESM.docx]

Journal of Nephrology

Exploring Biopsychosocial Correlates of Pregnancy Risk and Pregnancy Intention in Women with Chronic Kidney Disease

^1^Department of Women and Children’s Health, School of Life Course Sciences, King’s College London, United Kingdom.

^2^Department of Obstetric Medicine, Bart’s and the London NHS Foundation Trust, United Kingdom

^3^Department of Psychology, Institute of Psychiatry, Psychology and Neuroscience, King's College London, United Kingdom.

^4^Department of Renal Medicine, School of Inflammation, Immunology and Mucosal Biology, King’s College London, United Kingdom.

Correspondence: Elizabeth Ralston, King’s College London, 5th Floor Addison House, Guy’s Campus, London SE1 1UL. Email: Elizabeth.ralston@kcl.ac.uk

# Supplementary material

Table of Contents:

**Supplementary table 1**: Definition and data types of the demographic, pregnancy variables and psychological attributes collected

**Supplementary file. 1**: Assessing content validity of the perception of pregnancy risk questionnaire (PPRQ)

**Supplementary table 2.** Amendments made to the Perception of Pregnancy Risk Questionnaire.

**Supplementary table 3.** Comparison of demographic, psychological and clinical characteristics stratified by CKD stage.

**Supplementary table 4**. Describing the included data in multiple imputation with chained equations. The type of variable and method of applied imputation provided.

**Supplementary table 5**. Comparing the univariate relationships between individual variables and perception of pregnancy risk (outcome variable) with imputed data and complete case data.

**Supplementary table 6**. Comparison of adjusted regression models between imputed data and complete case data with perception of pregnancy risk as outcome variable.

**Supplementary table 7**. Comparing the univariate relationships between individual variables and pregnancy intention (outcome variable) with imputed data and complete case data

**Supplementary table 8**. Comparison of adjusted regression models between imputed data and complete case data with pregnancy intention as outcome variable.

**Supplementary table 9:** Pie chart illustrating the region where women were recruited from in the United Kingdom.

**Supplementary table 10:** Comparison between women who have received pre-pregnancy counselling versus those who have not.

**Supplementary table 11**: Pearson correlations between Kidney Disease Quality of Life Instrument and other psychological attributes measured

### Table. S1

Supplementary table 1: Definition and data types of the demographic, pregnancy variables and psychological attributes collected

| **Demographic and pregnancy preferences** | | |
| --- | --- | --- |
| **Variable** | **Description** | **Type** |
| Age | Age at participation | Numeric |
| Ethnicity | Identified ethnicity categorised | Categorical (White, Black, Asian, Mixed/Other) |
| Education | Highest educational qualification obtained | Categorical (GCSE/O-Level/Foundation diploma, A Level/International Baccalaureate/Advanced diploma, Undergraduate degree, Postgraduate degree and above) |
| Employment | Current employment status | Categorical (Full-time, Part-time, Homemaker, Retired, Unemployed, Student, Other). In main analysis binary indicator used (Employed; yes/no) |
| Living arrangement | Current living arrangement. | Categorical (Living with partner, living with relatives or friends, living alone) |
| Socio-economic status | Postcode was collected and Index of Multiple Deprivation Decile was calculated. This measures relative deprivation in each small area in England. | Numerical |
| Religion | Identified religion | Categorical (No religion, Christian, Buddhist, Hindu, Jewish, Muslim, Sikh, Other). In the main analysis a derived variable was used (Religion: yes/no) |
| Pre-pregnancy counselling clinic | An indicator variable whether the participant had previously attended a pre-pregnancy counselling clinic with a nephrologist | Binary |
| Importance of pregnancy to self | How important is pregnancy and childbearing to the participant. Responses were collected using a Likert scale from 1 being unimportant to 5 being very important. | Numeric |
| Importance of pregnancy to family | How important is pregnancy and childbearing to the participant’s family. Responses were collected using a Likert scale from 1 being unimportant to 5 being very important. | Numeric |
| Trying to conceive | An indication if the participant is currently trying to conceive, | Binary |
| Children | The number of children the participant has. | Numeric |
| Perceived CKD severity | How severe does the participant believe their CKD to be (visual analogue scale from 0 to 100). | Numeric |
| **Clinical characteristics** | | |
| Primary cause of CKD | The primary cause of their CKD | Categorical (Glomerulonephritis,  Chronic Progressive Nephropathy/ Vesicoureteral Reflux, Autosomal Dominant Polycystic Kidney Disease, Diabetes, Congenita/Inherited, Transplant, Other, Systemic Lupus Erythematosus, Unknown) |
| eGFR | The most recent eGFR (mls/min/1.73m^2^), calculated using CKD-EPI equation without ethnicity adjustment. | Numeric |
| Inpatient admission | The number of inpatient admissions the participant has had in the past five years. | Numeric |
| Previous dialysis | Has the participant ever had haemodialysis or peritoneal dialysis. | Binary |
| Previous transplant | Has the participant ever had a transplant. | Categorical (No not had a transplant, Yes had a transplant and it was pre-emptive, Yes had a transplant and it was not pre-emptive). In the analysis, a binary yes/no variable was used. |
| Chronic hypertension | Does the individual have a systolic blood pressure >140 mmHg, or a diastolic blood pressure >90 mmHg, or history of taking antihypertensives. | Binary |
| Clinically relevant proteinuria | Does the participant have an Albumin: creatinine ratio > 70 mg/mmol or Protein: creatinine ratio >100 mg/mmol. | Binary |

| **Psychological attributes** | |
| --- | --- |
| Perception of Pregnancy Risk Questionnaire (PPRQ) | The PPRQ is a nine-item scale with responses recorded on a visual analogue scale from 0 to 100. Results are reported as an overall mean score, and two subscale scores: Risk to Self and Risk to Baby. The greater the score, the greater the perceived pregnancy risk. |
| Desire to Avoid Pregnancy (DAP) | a 14-item scale which measures women’s prospective pregnancy preferences by three conceptual domains; 1) cognitive self-evaluation of preferences around pregnancy and childbearing, 2) affective feelings about a potential pregnancy and child, 3) anticipated practical consequences if pregnancy and childbearing were to happen. For application in this study a high score indicated stronger pregnancy intention and a lower score indicated stronger pregnancy avoidance. |
| Patient Health Questionnaire for Depression and Anxiety (PHQ-4) | This was used as a brief screening tool to measure anxiety and depression. The scale ranges from 0 to 12, with a higher score indicating greater anxiety and depression (distress). |
| Brief Illness Perceptions Questionnaire (B-IPQ) | This measured women’s illness perceptions which is their cognitive and emotional representations of their CKD. Eight-items are recorded on a ten-point Likert scale to give a total score (range 0 to 80). A higher score indicates a more negative illness beliefs. |
| Multidimensional Scale of Perceived Social Support (MSPSS) | This measures support from: significant others, friends, and family. Responses are collected on a seven-point Likert scale and a total mean score and mean scores for each subscale calculated. A higher score indicates greater perceived social support. |
| Kidney Disease Quality of Life Instrument Short Form (KDQOL) | This was used to measure quality of life, adapted for non-dialysis patients. A total of 76 items included i) kidney disease specific measures including symptom/problems, effects of kidney disease, burden of kidney disease, cognitive function, quality of social interaction, sexual function, sleep, social support, work status, patient satisfaction ii) 36-Item Health Survey ^1^ including physical function and limitations, emotional problems, pain, general health perceptions, social function, emotional well-being, and energy/fatigue. Three summary scores were calculated: Physical Component Score (PCS), Mental Component Score (MCS) and Kidney Disease Component Score (KDCS). The greater the score in these summary scores indicated a better health related quality of life (range 0 to 100). |
| COVID-19 Risk Perception Score | A mean score was calculated (range 0 to 7), the higher score indicating greater perceived coronavirus risk. |
| *Note*. CKD = chronic kidney disease; eGFR = Estimated glomerular filtration rate; CKD-EPI = Chronic Kidney Disease Epidemiology Collaboration 2009 equation | |

### SFile. 1

Assessing content validity of the perception of pregnancy risk questionnaire (PPRQ)

The content validity of the modified PPRQ was assessed, this aimed to determine whether the PPRQ was clear and representative and highlight the need to refine items to the PPRQ for non-pregnant women with CKD. Established methodology to assess content validity were followed ^2–4^. 21 experts from members of the Rare Renal Disease Clinical Study Group ‘Pregnancy and CKD’ and health psychologists from King’s College London reviewed the modified PPRQ. Each item on the modified PPRQ were assessed based upon their representativeness and clarity on a four-point Likert scale. Representativeness indicates how well the item fits the construct being measured and clarity is based upon whether the item is phrased clearly. Experts were also asked if the item should be included and whether the scale was comprehensive. An opportunity for experts to provide qualitative feedback was provided. If qualitative feedback was congruent, amendments were made to ensure the questionnaire was appropriate and sensitive for the population, especially considering women would be completing the survey remotely.

For both clarity and representativeness, the item content validity (I-CVI) was calculated and the content scale average (S-CVI/Av). I-CVI greater than 0.78 indicates strong conceptualisations of constructs and items ^5^. I-CVI was calculated by dividing the sum of experts who gave a Likert rating of 3 or 4 by the total number of experts ^4^. The content scale average (S-CVI/Av) was also calculated, this measures the overall representativeness or clarity of all nine PPRQ items. A value of greater than 0.90 was required for the measure to be valid ^6^. This was calculated by dividing the sum of I-CVI by the total number of items in the scale ^4^.

Each item of the modified PPRQ for representativeness achieved an I-CVI between 0.90 and 1, with an S-CVI/Av of 0.96. Both sub-scales maintaining strong representativeness (Risk to self: 0.95; Risk to baby: 0.97). Each item of the modified PPRQ for clarity received an I-CVI between 0.86 and 1 with and S-CVI/Av of 0.95, with both subscales maintaining strong clarity index (risk to self 0.96; risk to baby 0.95). All items remained included in the modified PPRQ as item inclusion ranged between 90% to 100% and lastly 100% of experts reported the PPRQ to be comprehensive. These results indicate the modified PPRQ is a valid instrument for assessing perception of pregnancy risk women in women with CKD outside of pregnancy. Several qualitative comments were provided and congruent, thus minor amendments were made. These amendments often included providing an example of risk and defining terminology (“Risk examples: low birth weight” and defining premature (before 37 weeks)).

The content validity of the amended PPRQ was then assessed again by five women with CKD in a pre-pregnancy counselling clinic at King’s College Hospital NHS Foundation trust. Each item of the amended PPRQ received an I-CVI between 0.80 and 1, with an S-CVI/Av of 0.91, both subscales continued to maintain strong content validity (risk to mother: 0.9 and risk to baby: 0.92). Item clarity also maintained an I-CVI between 0.8 and 1, with an S-CVI/Av of 0.96 (risk to mother 0.95 and risk to baby 0.96). Based upon the qualitative feedback from women minor amendments were made, this included replacing the word ‘defect’ and ‘dying’ when discussing the baby’s health. The results of the second content validity continue to demonstrate that the modified PPRQ is a valid instrument for assessing perceptions of pregnancy risk in women with CKD outside of pregnancy. Content validity was not assessed again as major amendments were not made. Table S2 reports the amendments made at each stage of the PRRQ. The research ethics committee requested minor amendments to be made to make it clear the items were addressing future pregnancy.

References:

1. Hays R, Sherbourne C, Mazel R: The Rand 36-item Health Survey 1.0. *Health Econ* 2: 217–227, 1993

2. Lynn M: Determination and Quantification Of Content Validity. *Nurs Res* 35: 1986

3. Rubio D, Berg-Weger M, Tebb S, Lee S, Rauch S: Objectifying content validity: Conducting a content validity study in social work research. *Soc Work Res* 27: 94-104., 2003

4. Polit D, Beck C: The Content Validity Index: Are You Sure You Know What’s Being Reported? Critique and Recommendations. *Res Nurs Health* 29: 489–497, 2006

5. Davis L: Instrument review: Getting the most from a panel of experts. *Applied Nursing Research* 5: 194–197, 1992

6. Waltz C, Strickland O, Lenz E, Soeken K: Validity of measures. In: *Measurement in nursing and health research*, 3rd ed., 2005

7. Heaman M, Gupton A: Psychometric testing of the perception of pregnancy risk questionnaire. *Res Nurs Health* 32: 493–503, 2009

| Table S2. Supplementary Table 2. Amendments made to the Perception of Pregnancy Risk Questionnaire. | | | |  |
| --- | --- | --- | --- | --- |
| **Original PPRQ** | **Version one PPRQ** | **Version two PPRQ** | **Version three PPRQ** | **Final modified PPRQ** |
| 1.The risk for myself during this pregnancy is: | 1. The risk to my kidneys during this pregnancy is: | 1. The risk of injury to my kidneys during this pregnancy is: | 1. The risk of injury to my kidneys during this pregnancy is: | 1. The risk of injury to my kidneys during a pregnancy is: |
| 2. The risk to my unborn baby during this pregnancy is | 2. The risk to my unborn baby during this pregnancy is | 2. The risk to my unborn baby, during pregnancy is:  Risk examples: Low birthweight or birth defects | 2. The risk to my unborn baby, during pregnancy is:  Risk examples: Low birthweight or cleft palate | 2. The risk to my unborn baby, during pregnancy is:  Risk examples: low birthweight or cleft palate |
| 3. My risk of hemorrhaging (losing too much blood) during this pregnancy is: | 3. My risk of hemorrhaging (losing too much blood) during this pregnancy is: | 3. My risk of losing too much blood (haemorrhaging) during this pregnancy is: | 3. My risk of losing too much blood (haemorrhaging) during this pregnancy is: | 3. My risk of losing too much blood (haemorrhaging) during a pregnancy is: |
| 4. My risk of having a caesarean section is: | 4. My risk of having a caesarean section is: | 4. My likelihood of having a caesarean section is: | 4. My likelihood of having a caesarean section is: | 4. My likelihood of having a caesarean section is |
| 5. My risk of dying during this pregnancy is: | 5. My risk of dying during this pregnancy is: | 5. The threat to my life during this pregnancy is: | 5. The threat to my life during this pregnancy is: | 5. The risk of losing my life during a pregnancy is: |
| 6. My baby’s risk of being born prematurely is: | 6. My baby’s risk of being born prematurely is: | 6. My baby’s risk of being born prematurely (before 37 weeks), is: | 6. My baby’s risk of being born prematurely (before 37 weeks), is: | 6. My future baby’s risk of being born prematurely (before 37 weeks), is |
| 7. My baby’s risk of having a birth defect is: | 7. My baby’s risk of having a birth defect is: | 7. My baby’s risk of having a birth defect is: | My baby’s risk of being born with a condition like Downs syndrome or congenital heart disease is: | 7. A future baby’s risk of being born with a condition like Downs syndrome or congenital heart disease is: |
| 8. My baby’s risk of needing to go to the Neonatal Intensive Care Unit is | 8. My baby’s risk of needing to go to the Neonatal Intensive Care Unit is | 8. My baby’s risk of needing to go to the Neonatal Care Unit is: | 8. My baby’s risk of needing to go to the Neonatal Care Unit is: | 8. A future baby’s risk of needing to go to the Neonatal Care Unit is: |
| 9. My baby’s risk of dying during this pregnancy is: | 9. My baby’s risk of dying during this pregnancy is: | 9. My baby’s risk of dying during this pregnancy is: | 9. My baby’s threat to life during this pregnancy is: | 9. The risk of losing a future baby during pregnancy is |
| *Note*. PPRQ = Perception of pregnancy risk questionnaire. Original is the original PPRQ ^7^. Version one is the is first adaption to make the PPRQ kidney disease specific and the version that 21 experts assessed its content validity. Version two reflects the congruent qualitative amendments provided by experts. Version three reflects the amendments from women with CKD. The final version includes the amendments recommended by the research ethics committee. | | | | |

### Table S3

Supplementary table 3. Comparison of demographic, psychological and clinical characteristics stratified by CKD stage.

|  | **Stage 1**  (eGFR > 90 ml/min/1.73m^2^) | **Stage 2**  (eGFR 60 – 89 ml/min/1.73m^2^) | **Stage 3a**  (eGFR 45 – 59 ml/min/1.73m^2^) | **Stage 3b**  (eGFR 30 – 44 ml/min/1.73m^2^) | **Stage 4**  (eGFR 15-29 ml/min/1.73m^2^) | **Stage 5**  (eGFR < 15 ml/min/1.73m^2^) | **p** |
| --- | --- | --- | --- | --- | --- | --- | --- |
| N | 84 | 80 | 44 | 48 | 38 | 17 |  |
| Age (mean (SD)) | 32.73 (7.16) | 35.21 (6.21) | 36.18 (7.34) | 36.85 (6.93) | 35.53 (7.47) | 36.71 (8.84) | 0.014 |
| Ethnicity (%) |  |  |  |  |  |  | 0.617 |
| Asian | 11 (13.3) | 7 (8.8) | 8 (18.2) | 5 (10.4) | 2 (5.3) | 2 (11.8) |  |
| Black | 11 (13.3) | 8 (10.0) | 7 (15.9) | 8 (16.7) | 8 (21.1) | 5 (29.4) |  |
| White | 51 (61.4) | 57 (71.2) | 23 (52.3) | 33 (68.8) | 27 (71.1) | 9 (52.9) |  |
| Mixed | 5 (6.0) | 3 (3.8) | 3 (6.8) | 1 (2.1) | 1 (2.6) | 0 (0.0) |  |
| Other | 5 (6.0) | 5 (6.2) | 3 (6.8) | 1 (2.1) | 0 (0.0) | 1 (5.9) |  |
| Education (%) |  |  |  |  |  |  | 0.142 |
| No formal education | 0 (0.0) | 1 (1.2) | 0 (0.0) | 2 (4.2) | 1 (2.6) | 0 (0.0) |  |
| GCSE (full time education to age 16) | 7 (8.3) | 15 (18.8) | 8 (18.2) | 16 (33.3) | 10 (26.3) | 4 (23.5) |  |
| A level (full time education to age 18) | 17 (20.2) | 17 (21.2) | 8 (18.2) | 6 (12.5) | 5 (13.2) | 5 (29.4) |  |
| Undergraduate degree | 35 (41.7) | 26 (32.5) | 16 (36.4) | 9 (18.8) | 13 (34.2) | 3 (17.6) |  |
| Postgraduate degree | 25 (29.8) | 21 (26.2) | 12 (27.3) | 15 (31.2) | 9 (23.7) | 5 (29.4) |  |
| Living arrangement (%) |  |  |  |  |  |  | 0.305 |
| Living with partner | 46 (55.4) | 54 (67.5) | 29 (65.9) | 38 (80.9) | 23 (60.5) | 9 (52.9) |  |
| Living with relatives/friends | 24 (28.9) | 14 (17.5) | 8 (18.2) | 5 (10.6) | 8 (21.1) | 4 (23.5) |  |
| Living alone | 13 (15.7) | 12 (15.0) | 7 (15.9) | 4 (8.5) | 7 (18.4) | 4 (23.5) |  |
| Socio-economic status^1^ (median (IQR)) | 5.00 [3.00, 7.00] | 5.00 [3.00, 8.00] | 5.00 [3.75, 7.25] | 5.00 [3.00, 8.00] | 4.00 [3.00, 6.25] | 4.00 [3.00, 6.00] | 0.859 |
| Actively practising religion -Yes (%) | 28 (33.3) | 12 (15.0) | 14 (31.8) | 17 (36.2) | 11 (29.7) | 7 (41.2) | 0.051 |
| Pregnancy importance (%) |  |  |  |  |  |  | 0.429 |
| Unimportant | 7 (8.3) | 5 (6.2) | 5 (11.4) | 8 (16.7) | 4 (10.5) | 2 (11.8) |  |
| Slightly important | 6 (7.1) | 5 (6.2) | 1 (2.3) | 1 (2.1) | 0 (0.0) | 1 (5.9) |  |
| Moderately important | 12 (14.3) | 8 (10.0) | 2 (4.5) | 3 (6.2) | 6 (15.8) | 4 (23.5) |  |
| Important | 14 (16.7) | 23 (28.7) | 13 (29.5) | 10 (20.8) | 9 (23.7) | 5 (29.4) |  |
| Very important | 45 (53.6) | 39 (48.8) | 23 (52.3) | 26 (54.2) | 19 (50.0) | 5 (29.4) |  |
| Importance of pregnancy to family (%) |  |  |  |  |  |  | 0.001 |
| Unimportant | 9 (10.7) | 4 (5.0) | 10 (22.7) | 9 (18.8) | 3 (7.9) | 3 (17.6) |  |
| Slightly important | 2 (2.4) | 6 (7.5) | 1 (2.3) | 0 (0.0) | 4 (10.5) | 4 (23.5) |  |
| Moderately important | 20 (23.8) | 12 (15.0) | 2 (4.5) | 8 (16.7) | 5 (13.2) | 1 (5.9) |  |
| Important | 21 (25.0) | 30 (37.5) | 11 (25.0) | 14 (29.2) | 17 (44.7) | 5 (29.4) |  |
| Very important | 32 (38.1) | 28 (35.0) | 20 (45.5) | 17 (35.4) | 9 (23.7) | 4 (23.5) |  |
| Perceived severity of kidney disease (0 to 100) (median (IQR)) | 26.00 [10.00, 40.00] | 40.00 [21.00, 50.00] | 51.50 [30.00, 67.25] | 60.00 [50.00, 73.00] | 72.00 [65.00, 80.00] | 83.00 [75.00, 100.00] | <0.001 |
| Attended pre-pregnancy counselling clinic = Yes (%) | 18 (21.4) | 26 (32.5) | 19 (43.2) | 25 (52.1) | 17 (44.7) | 2 (12.5) | 0.001 |
| eGFR^2^ (ml/min/1.73m^2^) (median (IQR)) | 108.00 [100.00, 118.00] | 74.50 [67.75, 80.43] | 54.00 [50.00, 56.25] | 37.00 [34.00, 41.00] | 22.00 [18.47, 26.75] | 10.00 [7.00, 12.00] | <0.001 |
| Previous dialysis = yes (%) | 4 (4.8) | 18 (22.5) | 6 (13.6) | 8 (17.4) | 6 (16.2) | 8 (47.1) | <0.001 |
| Previous transplant (%) |  |  |  |  |  |  | <0.001 |
| No | 79 (94.0) | 57 (71.2) | 23 (52.3) | 26 (55.3) | 25 (65.8) | 6 (35.3) |  |
| Yes – pre-emptive | 5 (6.0) | 16 (20.0) | 19 (43.2) | 16 (34.0) | 11 (28.9) | 7 (41.2) |  |
| Yes – not pre-emptive | 0 (0.0) | 7 (8.8) | 2 (4.5) | 5 (10.6) | 2 (5.3) | 4 (23.5) |  |
| Chronic hypertension^3^ **=** Yes (%) | 40 (52.6) | 33 (45.8) | 26 (61.9) | 30 (65.2) | 22 (62.9) | 14 (82.4) | 0.054 |
| Clinically relevant proteinuria^4^ = yes (%) | 9 (14.3) | 8 (17.4) | 8 (32.0) | 13 (34.2) | 11 (40.7) | 7 (70.0) | 0.001 |
| Perceived Pregnancy Risk ^5^ (mean (SD)) | 36.3 (20.6) | 43.4 (23.2) | 47.9 (23.1) | 52.7 (21.7) | 58.3 (21.3) | 60.3 (18.9) | <0.001 |
| Covid-19 Risk Perception^6^ (mean (SD)) | 4.7 (1.1) | 4.7 (0.9) | 4.8 (1.04) | 5.0 (1.1) | 5.0 (1.1) | 4.7 (1.0) | 0.441 |
| Pregnancy intention^7^ (median (IQR)) | 2.07 [1.21, 2.86] | 1.96 [1.21, 2.77] | 2.36 [1.25, 3.36] | 2.36 [1.07, 3.14] | 2.29 [1.36, 2.71] | 1.79 [0.86, 2.21] | 0.458 |
| Distress^8^ (median [IQR]) | 2.0 [1.0, 4.0] | 2.0 [0.0, 3.5] | 3.0 [0.0, 6.0] | 2.5 [0.75, 5.2] | 2.0 [0.2, 4.0] | 3.00 [1.0, 7.0] | 0.523 |
| Overall perceived social support ^9^ (median [IQR]) | 6.00 [5.2, 6.7] | 6.0 [5.2, 6.7] | 6.2 [5.3, 6.7] | 6.0 [5.1, 6.7] | 6.12 [5.3, 6.7] | 5.58 [5.3, 6.2] | 0.600 |
| Illness perceptions^10^ (mean (SD)) | 40.3 (13.2) | 45.2 (12.9) | 49.3 (12.7) | 51.77 (9.0) | 53.5 (9.6) | 58.41 (9.4) | <0.001 |
| Quality of Life^11^: Physical component summary (median [IQR]) | 75.9 [47.2, 87.5] | 81.3 [55.6, 87.5] | 80.6 [50.6, 86.2] | 75.3 [51.6, 83.4] | 68.8 [56.6, 83.1] | 45.0 [25.9, 70.8] | 0.012 |
| Quality of Life^11^: Mental component summary (median [IQR]) | 70.6 [42.5, 81.5] | 75.5 [55.1, 84.2] | 68.9 [36.5, 85.5] | 69.1 [55.5, 79.2] | 67.8 [55.8, 83.6] | 37.5 [29.0, 71.0] | 0.106 |
| Quality of Life^11^: Kidney disease component summary (mean (SD)) | 77.4 (15.4) | 78.8 (13.5) | 74.7 (15.3) | 73.8 (15.5) | 75.6 (15.4) | 68.9 (16.3) | 0.497 |
| *Note.* CKD stages defined according to the Kidney Disease Improving Global Outcomes. Abbreviations: eGFR = estimated glomerular filtration rate; CKD = chronic kidney disease; ^1^ Socioeconomic status is measured using the Index of Multiple Deprivation (IMD). The IMD measures relative deprivation across each small area in England in deciles, where 1 represents the most deprived 10% to 10 which represents the least deprived 10 percent. Deprivation is measures across seven domains: income, employment, education, health, crime, barriers to housing and services, and living environment. ^2^ Calculated using Chronic Kidney Disease Epidemiology Collaboration 2009 equation without ethnicity adjustment. ^3^History of antihypertensives and/or >140mmHg/ >90mmHg; ^4^Albumin:creatinine ratio > 70 mg/mmol or Protein: creatinine ratio > 100 mg/mmol. ^5^ Measured using a modified version of Perception of Pregnancy Risk Questionnaire (0 – 100).. ^6^ Measured using COVID-19 Risk Perception Score (1 - 7); ^7^Measured using Desire to Avoid Pregnancy Scale (0 – 4) ^8^Measured using Patient Health Questionnaire 4 Item Scale (0 – 12).^9^ Measured using Multidimensional Scale of Perceived Social Support (1 - 7). ^10^ Measured using Brief Illness Perceptions Questionnaire (0 - 78). ^11^ Measured using Kidney Disease Quality of Life Scale (0 - 100). | | | | | | | |

### Table. S4

Supplementary table 4 – Describing the included data in multiple imputation with chained equations. The type of variable and method of applied imputation provided.

| **Variable included** | **Type** | **Method applied for imputation** |
| --- | --- | --- |
| Age | Numeric | NA |
| Ethnicity | Factor (white, black, Asian, other) | Polytomous regression imputation |
| Education | Factor (No formal, GCSE, A Level, Undergrad, Postgrad) | NA |
| Employment | Binary (No / Yes) | Logistic regression |
| Living arrangement | Factor (Living with partner, living with family/friends, living alone) | Polytomous regression imputation |
| Socio-economic status^1^ | Numeric | Predictive mean matching |
| Religion | Binary (No/Yes) | Logistic regression |
| Active religion | Binary (No/Yes) | Logistic regression |
| Importance of pregnancy | Numeric | NA |
| Importance of pregnancy to family | Numeric | NA |
| Attended pre pregnancy counselling clinic | Binary (Y/N) | Logistic regression |
| Pregnancy history | Numeric | Predictive mean matching |
| Number of children | Numeric | Predictive mean matching |
| Perceived CKD severity | Numeric | Predictive mean matching |
| Pregnancy risk perception^2^ | Numeric | Predictive mean matching |
| Covid-19 Risk Perception^3^ | Numeric | Predictive mean matching |
| Pregnancy intention^4^ | Numeric | Predictive mean matching |
| Anxiety disorder^5^ | Numeric | Predictive mean matching |
| Depression^6^ | Numeric | Predictive mean matching |
| Perceived social support^7^ | Numeric | Predictive mean matching |
| Illness perception^8^ | Numeric | Predictive mean matching |
| Quality of life – Physical component summary^9^ | Numeric | Predictive mean matching |
| Quality of life – Mental component summary^9^ | Numeric | Predictive mean matching |
| Quality of life – kidney disease component summary^9^ | Numeric | Predictive mean matching |
| Cause of CKD | Factor (Glomerulonephritis, Chronic Progressive Nephropathy/ Vesicoureteral Reflux, Autosomal Dominant Polycystic Kidney Disease, Diabetes, Congenital/inherited, Transplant, Other/unknown, Systemic Lupus Erythematosus) | Polytomous regression imputation |
| No. of inpatient admissions in the past five years | Numeric | Predictive mean matching |
| Previous dialysis | Binary (Yes/No) | Logistic regression |
| Previous transplant | Factor (No, yes pre-emptive, yes not pre-emptive) | Polytomous regression imputation |
| eGFR^10^ | Numeric | Predictive mean matching |
| Clinically relevant proteinuria^11^ | Binary (Yes/No) | Logistic regression |
| Chronic hypertension^12^ | Binary (Yes/No) | Logistic regression |
| *Note.* Abbreviations: CKD = Chronic Kidney Disease; eGFR = estimated Glomerular Filtration Rate. ^1^Socioeconomic status is measured using the Index of Multiple Deprivation;  ^2^Overall score measured using a modified version of Perception of Pregnancy Risk Questionnaire; ^3^Overall score measured using COVID-19 Risk Perception Score; ^4^Overall score measured using Desire to Avoid Pregnancy; ^5^Overall score measured using Generalised Anxiety Disorder 2-item scale; ^6^Overall score measured using Patient Health Questionnaire 2 item scale; ^7^Overall score measured using Multidimensional Scale of Perceived Social Support; ^8^Overall score measured using Brief Illness Perceptions Questionnaire; ^9^Measured using Kidney Disease Quality of Life Scale; ^10^calculated using Chronic Kidney Disease Epidemiology Collaboration 2009 equation without ethnicity adjustment. ^11^ Albumin: creatinine ratio > 70 mg/mmol or Protein: creatinine ratio > 100 mg/mmol. ^12^ History of antihypertensives and/or >140mmHg/ >90mmHg | | |

### Table. S5

Supplementary table 5. Comparing the univariate relationships between individual variables and perception of pregnancy risk (outcome variable) with imputed data and complete case data.

|  | **Imputed data** |  | **Complete case** |  |
| --- | --- | --- | --- | --- |
| **Predictor** | **Coefficients** | ***p*** | **Coefficients** | ***p*** |
| Age | 0.5 (0.1 to 0.8) | 0.017 | 0.5 (0.1 to 0.8) | 0.015 |
| Ethnicity - Black | 1.5 (-6.0 to 8.9) | 0.70 | 1.1 (-6.7 to 8.8) | 0.79 |
| Ethnicity – Other | 3.8 (-5.7 to 13.4) | 0.43 | 4.6 (-4.9 to 14.2) | 0.34 |
| Ethnicity - Asian | 7.4 (-1.2 to 16.0) | 0.091 | 8.4 (-0.2 to 17.0) | 0.057 |
| Education - GCSE | -7.9 (-31.4 to 15.7) | 0.51 | -6.5 (-29.9 to 17.0) | 0.59 |
| Education – A level | -14.5 (-38.0 to 9.0) | 0.22 | -14.5 (-38.0 to 8.9) | 0.22 |
| Education - Undergraduate | -18.4 (-41.6 to 4.8) | 0.12 | -18.0 (-41.1 to 5.2) | 0.13 |
| Education - Postgraduate | -16.1 (-39.3 to 7.2) | 0.18 | -16.2 (-39.3 to 7.0) | 0.17 |
| Employed - Yes | -7.4 (-13.3 to 1.5) | 0.015 | -7.3 (-13.3 to 1.2) | 0.018 |
| Living arrangement – relatives/friends | -1.7 (-8.6 to 5.1) | 0.62 | -2.6 (-9.6 to 4.4) | 0.46 |
| Living arrangement - alone | 0.6 (-6.9 to 8.1) | 0.87 | -0.2 (-7.9 to 7.5) | 0.96 |
| Socioeconomic status^1^ | -0.7 (-1.8 to 0.3) | 0.18 | -0.9 (-2.0 to -0.1) | 0.086 |
| Religious - yes | 1.8 (-3.5 to 7.1) | 0.50 | 1.2 (-4.1 to 6.6) | 0.65 |
| Importance of pregnancy | 0.5 (-1.5 to 2.5) | 0.64 | 0.6 (-1.5 to 2.7) | 0.58 |
| Attended PPC | 8.9 (3.4 to 14.4) | 0.002 | 9.1 (3.7 to 14.6) | 0.001 |
| Importance of pregnancy to family | 0.1 (-1.9 to 2.1) | 0.92 | 0.2 (-1.8 to 2.3) | 0.82 |
| Pregnancy history | 1.3 (-0.2 to 2.8) | 0.08 | 1.3 (-0.2 to 2.8) | 0.098 |
| Children | 0.7 (-2.2 to 3.5) | 0.64 | 0.1 (-2.7 to 3.0) | 0.93 |
| Perceived CKD severity | 0.4 (0.3 to 0.5) | <0.001 | 0.4 (0.3 to 0.5) | <0.001 |
| **Clinical characteristics** | | | | |
| Previous dialysis - yes | 11.1 (4.0 to 18.1) | 0.002 | 10.8 (3.6 to 17.9) | 0.003 |
| Previous transplant - yes | 14.2 (8.7 to 19.7) | <0.001 | 13.5 (7.9 to 19.1) | <0.001 |
| eGFR^2^ | -0.2 (-0.3 to -0.2) | <0.001 | -0.2 (-0.3 to -0.2) | <0.001 |
| Clinically relevant proteinuria^3^ | 9.1 (1.8 to 16.4) | 0.016 | 10.7 (3.6-17.8) | 0.003 |
| Chronic hypertension^4^ | 7.9 (2.4 to 13.4) | 0.005 | 7.1 (1.7 to 12.6) | 0.011 |
| **Psychological characteristics** | | | | |
| Covid – risk perception^5^ | 5.2 (2.7 to 7.8) | <0.001 | 5.6 (3.1 to 8.1) | <0.001 |
| Pregnancy intention^6^ | -0.0 (-2.5 to 2.4) | 0.97 | 0.2 (-2.1 to 2.6) | 0.84 |
| Distress^7^ | 1.3 (0.5 to 2.2) | 0.002 | 1.3 (0.5 to 2.1) | 0.003 |
| Social support^8^ | -0.8 (-3.1 to 1.5) | 0.52 | -1.0 (-3.5 to 1.4) | 0.39 |
| Illness perceptions^9^ | 0.8 (0.6 to 1.0) | <0.001 | 0.8 (0.7 to 1.0) | <0.001 |
| Quality of life - physical^10^ | -0.2 (-0.3 to -0.0) | 0.006 | -0.1 (-0.3 to -0.0) | 0.008 |
| *Note.* Reported to one significant figure after decimal. Abbreviations: IMD = Index of Multiple Deprivation; PPC = pre-pregnancy counselling; eGFR = estimated glomerular filtration rate; CKD = chronic kidney disease. Reference categories: ethnicity = White ethnicity, education = no formal education, living arrangement = living with partner. ^1^Socioeconomic status is measured using the Index of Multiple Deprivation; ^2^eGFRcalculated using Chronic Kidney Disease Epidemiology Collaboration 2009 equation without ethnicity adjustment; ^3^Albumin:creatinine ratio > 70 mg/mmol or Protein: creatinine ratio > 100 mg/mmol; ^4^History of antihypertensives and/or >140mmHg/ >90mmHg; ^5^ Measured using COVID-19 Risk Perception Score; ^6^Measured using Desire to Avoid Pregnancy Scale; ^7^Measured using Patient Health Questionnaire 4 Item Scale; ^8^Measured using Multidimensional Scale of Perceived Social Support; ^9^Measured using Brief Illness Perceptions Questionnaire; ^10^Measured using Kidney Disease Quality of Life Scale physical component summary. | | | | |

### Table. S6

Supplementary table 6. Comparison of adjusted regression models between imputed data and complete case data with perception of pregnancy risk as outcome variable.

|  |  | **Imputed data results** | | | | **Complete case results** | | | |
| --- | --- | --- | --- | --- | --- | --- | --- | --- | --- |
|  |  | **Model 1** |  | **Model 2** |  | **Model 1** |  | **Model 2** |  |
|  |  | **Coefficients** | **p** | **Coefficients** | **p** | **Coefficients** | **p** | **Coefficients** | **p** |
|  | (Intercept) | 46.5 (30.4 to 62.5) | <0.001 | -7.9 (-33.5 to 17.6) | 0.54 | 47.2 (25.4 to 69.1) | <0.001 | -16.6 (-48.9 to 15.8) | 0.31 |
| Demographic factors | Age | 0.1 (-0.2 to 0.5) | 0.43 | 0.1 (-0.3 to 0.4) | 0.68 | 0.2 (-0.3 to 0.7) | 0.39 | 0.2 (-0.3 to 0.7) | 0.37 |
|  | Employed | -6.1 (-11.6 to -0.7) | 0.028 | -3.7 (-9.1 to 1.7) | 0.17 | -9.0 (-15.7 to -2.4) | 0.008 | -6.8 (-13.5 to -0.1) | 0.05 |
|  | Attended PPC | 6.8 (1.6 to 12.0) | 0.011 | 6.5 (1.5 to 11.4) | 0.01 | 3.5 (-2.7 to 9.7) | 0.27 | 4.8 (-1.2 to 10.9) | 0.11 |
| Clinical characteristics | eGFR^1^ | -0.2 (-0.2 to -0.1) | <0.001 | -0.03 (-0.1 to 0.1) | 0.49 | -0.1 (-0.2 to -0.04) | 0.008 | -0.03 (-0.1 to 0.1) | 0.60 |
|  | Previous dialysis | 3.5 (-4.2 to 11.2) | 0.37 | 1.5 (-5.7 to 8.8) | 0.68 | -2.7 (-12.9 to 7.6) | 0.61 | -4.8 (-14.5 to 4.9) | 0.33 |
|  | Previous transplant | 7.8 (1.4 to 14.3) | 0.017 | 4.7 (-1.5 to 10.9) | 0.14 | 7.5 (-0.8 to 15.9) | 0.076 | 0.3 (-7.8 to 8.3) | 0.95 |
|  | Clinically relevant proteinuria^2^ - yes | 3.9 (-3.9 to 11.6) | 0.33 | 5.0 (-2.9 to 12.9) | 0.21 | 5.4 (-2.0 to 12.9) | 0.15 | 6.8 (-0.1 to 13.6) | 0.05 |
|  | Chronic hypertension^3^ | 4.2 (-1.2 to 9.6) | 0.13 | 2.0 (-3.3 to 7.3) | 0.47 | 4.1 (-2.4 to 10.6) | 0.21 | 3.2 (-3.2 to 9.6) | 0.32 |
| Psychological attributes | Covid risk perception^4^ | - | - | 2.7 (0.3 to 5.2) | 0.031 | - | - | 2.2 (-0.8 to 5.2) | 0.14 |
|  | Distress^5^ | - | - | 0.7 (-0.2 to 1.6) | 0.14 | - | - | 0.02 (-1.0 to 1.0) | 0.97 |
|  | Illness perceptions^6^ | - | - | 0.4 (0.2 to 0.7) | 0.001 | - | - | 0.6 (0.2 to 0.9) | 0.00 |
|  | Quality of life - physical^7^ | - | - | 0.1 (-0.03 to 0.2) | 0.13 | - | - | 0.2 (0.003 to 0.3) | 0.05 |
|  | Perceived CKD severity | - | - | 0.1 (0.04 to 0.3) | 0.010 | - | - | 0.17 (0.03 to 0.3) | 0.02 |
| Model summary | R^2^ | 0.21 | - | 0.33 | - | 0.20 |  | 0.36 |  |
|  | ΔR^2^ | 0.19 | - | 0.30 | - | 0.17 |  | 0.31 |  |
| *Note*. Reported to one significant figure after decimal. Model 1 adjusts for demographic and clinical factors only. Model 2 adjusts for demographic, clinical and psychological factors. Abbreviations: PPC = pre-pregnancy counselling; eGFR = estimated glomerular filtration rate; CKD = chronic kidney disease; R^2^ = R squared; ΔR^2^ = Adjusted R square. ^1^eGFR calculated using Chronic Kidney Disease Epidemiology Collaboration 2009 equation without ethnicity adjustment; ^2^Albumin:creatinine ratio > 70 mg/mmol or Protein: creatinine ratio > 100 mg/mmol; ^3^ History of antihypertensives and/or >140mmHg/ >90mmHg; ^4^ Measured using COVID-19 Risk Perception Score; ^5^Measured using Patient Health Questionnaire 4 Item Scale; ^6^ Measured using Brief Illness Perceptions Questionnaire; ^7^ Measured using Kidney Disease Quality of Life Scale - physical component summary. | | | | | | | | | |

### Table. S7

Supplementary table 7. Comparing the univariate relationships between individual variables and pregnancy intention (outcome variable) with imputed data and complete case data

| **Univariate analyses** | | | | |
| --- | --- | --- | --- | --- |
|  | **Imputed data** | | **Complete data** | |
| **Predictor** | **Coefficients** | **p** | **Coefficients** | **p** |
| Age | -0.01 (-0.03 to 0.01) | 0.22 | -0.01 (-0.03 to 0.01) | 0.29 |
| Ethnicity - Black | 0.4 (0.1 to 0.7) | 0.026 | 0.4 (0.02 to 0.7) | 0.039 |
| Ethnicity – Other | 0.2 (-0.3 to 0.6) | 0.48 | 0.1 (-0.3 to 0.6) | 0.50 |
| Ethnicity - Asian | 0.9 (0.5 to 1.3) | <0.001 | 1.0 (0.6 to 1.4) | <0.001 |
| Education - GCSE | -0.2 (-1.4 to 0.9) | 0.73 | -0.2 (-1.4 to 0.9) | 0.72 |
| Education – A level | -0.02 (-1.2 to 1.1) | 0.97 | -0.03 (-1.2 to 1.1) | 0.96 |
| Education - Undergraduate | -0.2 (-1.3 to 1.0) | 0.79 | -0.2 (-1.3 to 1.0) | 0.79 |
| Education - Postgraduate | -0.2 (-1.3 to 1.0) | 0.78 | -0.1 (-1.3 to 1.0) | 0.84 |
| Employed - Yes | 0.1 (-0.2 to 0.4) | 0.62 | 0.1 (-0.2 to 0.4) | 0.61 |
| Living arrangement – relatives/friends | -0.3 (-0.6 to 0.0) | 0.072 | -0.3 (-0.6 to 0.01) | 0.055 |
| Living arrangement - alone | -0.3 (-0.7 to 0.1) | 0.089 | -0.3 (-0.7 to 0.01) | 0.06 |
| Socio-economic status^1^ | -0.0 (-0.1 to 0.0) | 0.085 | -0.05 (-0.1 to 0.003) | 0.007 |
| Religious - yes | 0.5 (0.3 to 0.8) | <0.001 | 0.5 (0.3 to 0.8) | <0.001 |
| Importance of pregnancy | 0.4 (0.3 to 0.5) | <0.001 | 0.4 (0.3-0.5) | <0.001 |
| Attended PPC | 0.6 (0.3 to 0.8) | <0.001 | 0.6 (0.3 to 0.9) | <0.001 |
| Importance of pregnancy to family | 0.3 (0.2 to 0.4) | <0.001 | 0.3 (0.2 to 0.4) | <0.001 |
| Pregnancy history | -0.0 (-0.1 to 0.0) | 0.27 | -0.04 (-0.1 to 0.03) | 0.254 |
| Children | -0.2 (-0.4 to -0.1) | <0.001 | -0.2 (-0.4 to -0.1) | <0.001 |
| Perceived CKD severity | 0.002 (-0.01 to 0.003) | 0.51 | -0.002(-0.006 to 0.003) | 0.44 |
| **Clinical characteristics** | | | | |
| Previous dialysis - yes | 0.1 (-0.3 to 0.4) | 0.65 | 0.1 (-0.3 to 0.4) | 0.74 |
| Previous transplant - yes | 0.1 (-0.2 to 0.3) | 0.70 | 0.1 (-0.2 to 0.3) | 0.62 |
| eGFR^2^ | 0.0001 (-0.004 to 0.004) | 0.97 | 0.00002 (-0.004 to 0.004) | 0.99 |
| Clinically relevant proteinuria^3^ | -0.1 (-0.4 to 0.3) | 0.64 | 0.1 (-0.1 to 0.4) | 0.36 |
| Chronic hypertension^4^ | 0.1 (-0.1 to 0.4) | 0.29 | -0.1 (-0.1 to 0.33) | 0.36 |
| **Psychological characteristics** | | | | |
| Covid – risk perception^5^ | -0.2 (-0.4 to -0.1) | <0.001 | -0.2 (-0.4 to -0.1) | <0.001 |
| Perception of pregnancy risk^6^ | 0.0001 (-0.01 to 0.01) | 0.97 | 0.001 (-0.01 to 0.01) | 0.84 |
| Distress^7^ | -0.1 (-0.1 to -0.01) | 0.009 | -0.05 (-0.1 to -0.01) | 0.017 |
| Social support^8^ | 0.1 (-0.03 to 0.2) | 0.15 | 0.1 (-0.03 to 0.2) | 0.13 |
| Illness perceptions^9^ | -0.004 (-0.01 to 0.01) | 0.39 | -0.004 (-0.013 to 0.006) | 0.47 |
| Quality of life – physical^10^ | 0.01 (0.001 to 0.01) | 0.013 | 0.006 (0.001 to 0.01) | 0.02 |
| *Note*. Reported to one significant figure after decimal. Abbreviations: IMD = Index of Multiple Deprivation; PPC = Pre-Pregnancy Counselling; eGFR = estimated Glomerular Filtration Rate; CKD = Chronic Kidney Disease. Reference categories: ethnicity = White ethnicity, education = no formal education, living arrangement = living with partner. ^1^Socioeconomic status is measured using the Index of Multiple Deprivation; ^2^eGFRcalculated using Chronic Kidney Disease Epidemiology Collaboration 2009 equation without ethnicity adjustment; ^3^Albumin:creatinine ratio > 70 mg/mmol or Protein: creatinine ratio > 100 mg/mmol ^4^History of antihypertensives and/or >140mmHg/ >90mmHg; ^5^ Measured using COVID-19 Risk Perception Score; ^6^Measured using a modified version of Perception of Pregnancy Risk Questionnaire; ^7^Measured using Patient Health Questionnaire 4 Item Scale; ^8^ Measured using Multidimensional Scale of Perceived Social Support; ^9^Measured using Brief Illness Perceptions Questionnaire; ^10^Measured using Kidney Disease Quality of Life Scale physical component summary. | | | | |

### Table. S8

Supplementary table 8. Comparison of adjusted regression models between imputed data and complete case data with pregnancy intention as outcome variable.

|  |  | **Imputed data** | | | | **Completed case** | | | |
| --- | --- | --- | --- | --- | --- | --- | --- | --- | --- |
|  |  | **Model 1** |  | **Model 2** |  | **Model 1**  **(N = 194)** |  | **Model 2**  **(N = 182)** |  |
|  | **Variable** | **Coefficients** | **P** | **Coefficients** | **p** | **Coefficients** | **P** | **Coefficients** | **p** |
|  | (Intercept) | 0.4 (-0.1 to 0.9) | 0.11 | 0.9 (0.1 to 1.7) | 0.035 | 0.4 (-0.2 to 1.0) | 0.18 | 0.6 (-0.4 to-1.7) | 0.22 |
| Demographic factors | Ethnicity: Black | 0.02 (-0.3 to 0.4) | 0.89 | -0.003 (-0.3 to 0.3) | 0.99 | 0.1 (-0.3 to 0.5) | 0.55 | 0.1 (-0.3 to 0.5) | 0.55 |
|  | Ethnicity: Other | 0.01 (-0.4 to 0.4) | 0.94 | 0.1 (-0.3 to 0.5) | 0.69 | 0.1 (-0.3 to 0.5) | 0.63 | 0.1 (-0.3 to 0.5) | 0.68 |
|  | Ethnicity: Asian | 0.3 (-0.1 to 0.7) | 0.13 | 0.3 (-0.1 to 0.7) | 0.13 | 0.3 (-0.1 to 0.7) | 0.19 | 0.4 (-0.04 to 0.9) | 0.072 |
|  | Religion - yes | 0.3 (0.05 to 0.5) | 0.019 | 0.3 (0.03 to 0.5) | 0.028 | 0.3 (0.02 to 0.6) | 0.038 | 0.3 (-0.1 to 0.6) | 0.11 |
|  | Importance of pregnancy | 0.4 (0.2 to 0.5) | <0.001 | 0.4 (0.2 to 0.5) | <0.001 | 0.3 (0.2 to 0.5) | <0.001 | 0.4 (0.2 to 0.5) | <0.001 |
|  | Attended PPC | 0.3 (0.05 to 0.5) | 0.019 | 0.3 (0.01 to 0.5) | 0.041 | 0.3 (0.01 to 0.6) | 0.039 | 0.3 (-0.02-0.6) | 0.069 |
|  | Importance of pregnancy to family | 0.01 (-0.1 to 0.1) | 0.87 | 0.0 (-0.1 to 0.2) | 0.76 | 0.1 (-0.1 to 0.2) | 0.42 | 0.1 (-0.1 to 0.2) | 0.44 |
|  | Children | -0.3 (-0.4 to -0.2) | <0.001 | -0.3 (-0.4 to -0.2) | <0.001 | -0.4 (-0.5 to -0.2) | <0.001 | -0.4 (-0.5 to -0.2) | <0.001 |
| Clinical characteristics | eGFR^1^ | 0.0002 (-0.003 to 0.004) | 0.92 | 0.0005 (-0.004 to 0.003) | 0.79 | -0.0001 (-0.004 to 0.004) | 0.97 | -0.001 (-0.01 to 0.04) | 0.74 |
|  | Previous dialysis - yes | 0.3 (-0.1 to 0.6) | 0.12 | 0.3 (-0.1 to 0.6) | 0.096 | 0.3 (-0.1 to 0.8) | 0.13 | 0.4 (-0.1 to 0.9) | 0.075 |
|  | Previous transplant - yes | 0.02 (-0.3 to 0.3) | 0.87 | 0.005 (-0.3 to 0.3) | 0.97 | -0.01 (-0.4 to 0.3) | 0.97 | -0.1 (-0.5 to 0.3) | 0.67 |
|  | Clinically relevant proteinuria^2^ - yes | -0.2 (-0.5 to 0.1) | 0.11 | -0.2 (-0.5 to 0.1) | 0.11 | -0.2 (-0.5 to 0.2) | 0.36 | -0.2 (-0.5 to 0.1) | 0.27 |
|  | Chronic hypertension^3^ – yes | 0.2 (-0.1 to 0.4) | 0.16 | 0.2 (-0.001 to 0.5) | 0.051 | 0.03 ( -0.3 to 0.3) | 0.84 | 0.1 (-0.2 to 0.4) | 0.64 |
| Psychological attributes | Covid risk perception^4^ | - | - | -0.1 (-0.2 to -0.01) | 0.029 | - | - | -0.1 (-0.2 to 0.1) | 0.40 |
|  | Quality of life - physical^5^ | - | - | 0.003 (-0.002 to 0.01) | 0.28 | - | - | 0.001 (-0.01 to 0.01) | 0.73 |
|  | Distress^6^ | - | - | 0.02 (-0.1 to 0.02) | 0.25 |  |  | -0.01 (-0.1 to 0.04) | 0.66 |
| Model summary | R^2^ | 0.33 |  | 0.36 |  | 0.40 |  | 0.41 |  |
|  | ΔR^2^ | 0.30 |  | 0.33 |  | 0.35 |  | 0.36 |  |
| *Note.* Reported to one significant figure after decimal. Model 1 adjusts for demographic and clinical factors only. Model 2 adjusts for demographic, clinical and psychological factors. Abbreviations: PPC = pre-pregnancy counselling; eGFR = estimated glomerular filtration rate; CKD = chronic kidney disease; R^2^ = R squared; ΔR^2^ = Adjusted R square. ^1^eGFR calculated using Chronic Kidney Disease Epidemiology Collaboration 2009 equation without ethnicity adjustment; ^2^Albumin:creatinine ratio > 70 mg/mmol or Protein: creatinine ratio > 100 mg/mmol; ^3^ History of antihypertensives and/or >140mmHg/ >90mmHg; ^4^ Measured using COVID-19 Risk Perception Score; ^5^ Measured using Kidney Disease Quality of Life Instrument Short Form - physical component summary; ^6^Measured using Patient Health Questionnaire 4 Item Scale. | | | | | | | | | |

### Table S9.

Supplementary table 9: Pie chart illustrating the region where women were recruited from in the United Kingdom.
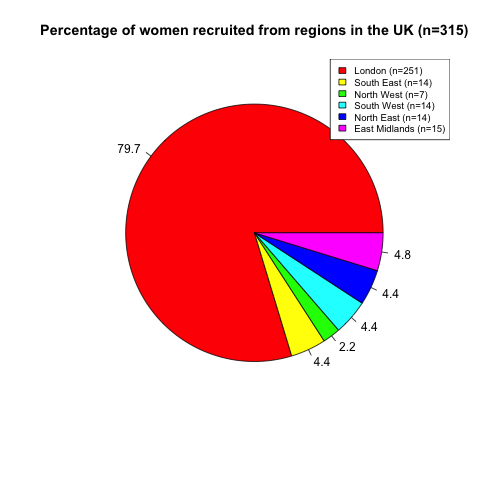


### Table S10.

Supplementary table 10: Comparison between women who have received pre-pregnancy counselling versus those who have not.

|  | No – have not received pre-pregnancy counselling | Yes - previously received pre-pregnancy counselling | *p* |
| --- | --- | --- | --- |
| N | 206 | 108 |  |
| Age (years) (mean (SD)) | 34.33 (7.57) | 36.33 (6.05) | 0.018 |
| Ethnicity (%) |  |  | 0.001 |
| Asian | 13 (6.3) | 23 (21.3) |  |
| Black | 31 (15.1) | 16 (14.8) |  |
| White | 140 (68.3) | 62 (57.4) |  |
| Mixed | 12 (5.9) | 1 (0.9) |  |
| Other | 9 (4.4) | 6 (5.6) |  |
| Education (%) |  |  | 0.001 |
| No formal education | 3 (1.5) | 1 (0.9) |  |
| GCSE (full time education to age 16) | 44 (21.4) | 17 (15.7) |  |
| A Level (full time education to age 18) | 47 (22.8) | 13 (12.0) |  |
| Undergraduate degree | 70 (34.0) | 32 (29.6) |  |
| Postgraduate degree | 42 (20.4) | 45 (41.7) |  |
| Living arrangement (%) |  |  | 0.017 |
| Living with partner | 122 (59.5) | 80 (74.8) |  |
| Living with relatives/friends | 50 (24.4) | 13 (12.1) |  |
| Living alone | 33 (16.1) | 14 (13.1) |  |
| Socio-economic status^1^ (median [IQR]) | 4.50 [3.0, 7.0] | 5.0 [3.0, 8.0] | 0.438 |
| Actively practising religion - yes (%) | 53 (25.7) | 36 (34.0) | 0.164 |
| Importance of pregnancy (%) |  |  | 0.002 |
| Unimportant | 28 (13.6) | 3 (2.8) |  |
| Slightly important | 13 (6.3) | 1 (0.9) |  |
| Moderately important | 23 (11.2) | 12 (11.1) |  |
| Important | 50 (24.3) | 25 (23.1) |  |
| Very important | 92 (44.7) | 67 (62.0) |  |
| Perceived severity of kidney disease (0 to 100) (median [IQR]) | 47.0 [22.0, 65.0] | 51.0 [33.5, 70.0] | 0.025 |
| eGFR^2^ (ml/min/1.73m^2^) (median [IQR]) | 68.0 [40.5, 101.50] | 54.0 [35.0, 80.0] | 0.007 |
| Previous dialysis - yes (%) | 35 (17.1) | 14 (13.2) | 0.470 |
| Previous transplant (%) |  |  | 0.398 |
| No | 150 (72.8) | 70 (65.4) |  |
| Yes – pre-emptive | 44 (21.4) | 28 (27.1) |  |
| Yes – not pre-emptive | 12 (5.8) | 8 (7.5) |  |
| Chronic hypertension^3^ - yes (%) | 109 (56.8) | 58 (58.6) | 0.864 |
| Clinically relevant proteinuria^4^ – yes (%) | 36 (28.1) | 20 (24.7) | 0.700 |
| CKD cause (%) |  |  | 0.192 |
| Glomerulonephritis | 40 (20.2) | 24 (22.9) |  |
| Chronic Progressive Nephropathy/ Vesicoureteral Reflux | 29 (14.6) | 21 (20.0) |  |
| Autosomal Dominant Polycystic Kidney Disease | 36 (18.2) | 11 (10.5) |  |
| Diabetic nephropathy | 19 (9.6) | 5 (4.8) |  |
| Congenital / inherited | 18 (9.1) | 12 (11.4) |  |
| Transplant | 3 (1.5) | 6 (5.7) |  |
| Other | 27 (13.6) | 11 (10.5) |  |
| Systemic Lupus Erythematosus | 22 (11.1) | 13 (12.4) |  |
| Unknown | 4 (2.0) | 2 (1.9) |  |
| Perceived Pregnancy Risk^5^ (mean (SD)) | 42.54 (23.74) | 51.67 (21.35) | 0.001 |
| Pregnancy intention^6^ (median [IQR]) | 1.86 [1.00, 2.71] | 2.57 [1.64, 3.36] | <0.001 |
| Quality of life^7^ – physical (median [IQR]) | 71.88 [45.00, 85.00] | 80.00 [59.69, 87.50] | 0.026 |
| Illness perceptions^8^ (mean (SD)) | 46.38 (13.71) | 48.42 (11.88) | 0.194 |
| Social support^9^ (median [IQR]) | 6.00 [5.15, 6.67] | 6.17 [5.54, 6.75] | 0.067 |
| Distress^10^ (median [IQR]) | 3.00 [0.00, 5.00] | 2.00 [0.00, 4.00] | 0.048 |
| Note. Abbreviations: eGFR = estimated glomerular filtration rate; CKD = chronic kidney ^1^Socioeconomic status is measured using the Index of Multiple Deprivation (IMD). The IMD measures relative deprivation across each small area in England in deciles, where 1 represents the most deprived 10% to 10 which represents the least deprived 10 percent. Deprivation is measures across seven domains; income, employment, education, health, crime, barriers to housing and services, and living environment. ^2^ eGFR calculated using Chronic Kidney Disease Epidemiology Collaboration 2009 equation without ethnicity adjustment; ^3^History of antihypertensives and/or >140mmHg/ >90mmHg; ^4^Albumin:creatinine ratio > 70 mg/mmol or Protein: creatinine ratio > 100 mg/mmol; ^5^ Measured using a modified version of Perception of Pregnancy Risk Questionnaire, range 0 to 100; ^6^Measured using Desire to Avoid Pregnancy Scale, range 0 to 4. ^7^Measured using Kidney Disease Quality of Life Scale – physical component summary, range 0 to 100; ^8^Measured using Brief Illness Perceptions Questionnaire, range 0 to 78; ^9^Measured using Multidimensional Scale of Perceived Social Support, range 1 to 7; ^10^Measured using Patient Health Questionnaire 4 Item Scale, range 0 to 12. | | | |

### Table. S11

Supplementary table 11 Pearson correlations between Kidney Disease Quality of Life Instrument and other psychological attributes measured

|  |  | Kidney disease component summary | Physical component summary | Mental component summary | Covid risk perception^1^ | Pregnancy intention^2^ | Distress^3^ | Social support^4^ | Illness perception^5^ | Perception of pregnancy risk^6^ |
| --- | --- | --- | --- | --- | --- | --- | --- | --- | --- | --- |
| **Kidney Disease Quality of Life Instrument subscales** | Kidney disease component summary | - | 0.79 | 0.76 | -0.21 | 0.08 | -0.66 | 0.38 | -0.52 | -0.29 |
|  | Physical component summary |  | - | 0.75 | -0.20 | 0.14 | -0.50 | 0.27 | -0.49 | -0.16 |
|  | Mental component summary |  |  | - | -0.24 | 0.18 | -0.75 | 0.32 | -0.35 | -0.14 |
| *Note.* ^1^Measured using COVID-19 Risk Perception Score; ^2^Measured using Desire to Avoid Pregnancy Scale; ^3^Measured using Patient Health Questionnaire 4 Item Scale; ^4^Measured using Multidimensional Scale of Perceived Social Support; ^5^Measured using Brief Illness Perceptions Questionnaire; ^6^Measured using a modified version of Perception of Pregnancy Risk Questionnaire; ^7^Measured using Kidney Disease Quality of Life Instrument Short Form | | | | | | | | | | |
